# A novel data-driven NLMPC strategy for techno-economic microgrid management with battery energy storage under uncertainty

**DOI:** 10.1038/s41598-025-13906-3

**Published:** 2025-08-01

**Authors:** Elnaz Yaghoubi, Elaheh Yaghoubi, Mehdi Zareian Jahromi, Mohammad Reza Maghami, Ali Paşaoğlu, Harold R. Chamorro

**Affiliations:** 1 Faculty of Engineering, Department of Electrical and Electronics Engineering, Karabuk University, Karabuk, Turkey; 2 Department of Electrical Engineering, Amirkabir University of Technology (Tehran-Polytechnic), Tehran, Iran; 3 Strategic Research Institute (SRI), Asia Pacific University of Technology and Innovation (APU), Jalan Teknologi 5, Kuala Lumpur, 57000 Malaysia; 4 Engineering and Natural Science Faculty, Computer Engineering Dept, İstanbul Rumeli University, İstanbul, Turkey; 5 Department of Electric Power Systems, KTH Royal Institute of Technology, Stockholm, Sweden

**Keywords:** Nonlinear MPC, Real-time, Energy storage, Microgrid, Data-driven, Uncertainty, Electrical and electronic engineering, Energy infrastructure

## Abstract

As renewable energy sources become more widespread and energy consumption continues to grow, there is an urgent requirement for smarter, more flexible control methods to manage microgrids (MGs) effectively. This study proposes a data-driven nonlinear model predictive control (NLMPC) framework for optimized MG operation, emphasizing energy storage system (ESS) integration. Effective MG management is crucial given increasing renewable penetration and energy demands. This framework coordinates distributed generation (DG) units, including rotating and non-rotating resources, with a battery ESS in a dynamic MG environment. Leveraging Gaussian Process Regression (GPR), the framework accurately models the complex dynamics of both DG units and the ESS. Unlike traditional model-based approaches, GPR learns system behavior from operational data, enabling precise performance prediction under varying conditions. This accuracy is crucial for optimized resource dispatch and efficient MG operation. GPR models capture ESS charging/discharging characteristics, efficiency, and state-of-charge (SOC) dynamics for informed ESS utilization. To address renewable energy uncertainties, Monte Carlo simulations are incorporated. This allows robust evaluation of the control strategy under various scenarios, ensuring MG stability and reliability despite fluctuating renewable generation. By considering these uncertainties, the NLMPC controller proactively manages DG and ESS dispatch, mitigating forecast errors and maximizing renewable energy use. The framework aims to achieve optimal power flow, balancing supply and demand while respecting operational constraints. This includes constraints on DG units, the ESS (SOC limits, charge/discharge rates), and overall MG operation (voltage and frequency stability). The NLMPC controller dynamically adjusts DG and ESS setpoints to minimize costs, maximize renewable energy use, and ensure MG stability and reliability. Simulation results demonstrate the framework’s effectiveness. Significant cost savings (approximately 39.2% compared to Conventional MPC and 41.5% compared to Adaptive MPC) and voltage stability improvements (28.57% and 52.38% respectively) are achieved. These improvements stem from accurate system dynamics modeling, robust uncertainty handling, and coordinated DG and ESS control.

## Introduction

### Motivation and importance

The rapid expansion of renewable energy sources (RES) and DG units in modern power systems has necessitated the development of advanced control strategies for the efficient operation of MG^1,2^. This shift has led to the development of MGs, which operate as restricted energy systems capable of functioning both independently and in conjunction with the main grid^3^. MGs offer many advantages, including enhanced energy security, improved power quality, and the ability to incorporate sustainable energy resources^4–6^. However, the inherent variability of RESs, coupled with varying load demands, poses significant operational challenges for MGs^7,8^. With MGs incorporating a mix of rotating and non-rotating energy resources, achieving stability, cost-efficiency, and operational resilience emerges as a paramount challenge^9,10^. In this context, implementing robust and adaptive control frameworks is crucial for achieving techno-economic optimization and real-time decision-making^11,12^. NLMPC has emerged as a powerful control method for managing the complexities associated with MG operations. Unlike traditional linear control methods, NLMPC can handle nonlinear system dynamics, operational constraints, and multi-objective optimization, making it well-suited for controlling hybrid MGs^13^. Moreover, the integration of data-driven approaches with NLMPC can significantly enhance operational efficiency by using real-time data for predictive and informed decision-making^14^. This hybrid approach allows MGs to maintain stability, optimize energy utilization, and ensure cost-effective operations^15^. Uncertainty is a vital factor in MG operation due to variable weather conditions, fluctuating load demands, and unpredictable market price changes^16^. Overlooking these uncertainties can lead to poor decision-making, energy imbalances, and higher operational costs^17^. Thus, robust control frameworks that incorporate predictive models are necessary to ensure the techno-economic efficiency of MGs^18^. Data-driven NLMPC fulfills this need by utilizing machine learning techniques and real-time data to improve predictions and adjust control actions dynamically^19^. This adaptive capability enables MGs to maintain resilient and cost-effective operations, even amidst uncertainty^20^.

### Literature review

The integration of MGs into modern power systems is essential for enhancing energy resilience, efficiency, and sustainability^21,22^. Advanced control methods have paved the way for NLMPC to emerge as a promising solution for optimizing MG operations^23^. Given the uncertainties in energy generation and consumption, data-driven approaches have become vital for enabling more adaptive and robust control^24^. The primary objective of optimizing MGs is to balance supply and demand while minimizing operational costs and enhancing system reliability^25^. As a model-based control strategy, NLMPC requires solving an optimization problem at each control interval and predicting future system behavior to determine the optimal control action^26^. Unlike linear MPC, NLMPC can manage nonlinear system dynamics, making it ideal for MGs with nonlinear power flow characteristics^27^. The availability of extended datasets from smart meters, sensors, and IoT devices has facilitated the implementation of data-driven control approaches^28^. Machine learning and deep learning models are applied to achieve high-accuracy forecasting and develop predictive models for the load demand and the generation rates of RESs^29^. Uncertainty in MG operations arises from the variability of RESs, fluctuations in load demand, and changes in energy market prices^30^. To tackle these challenges, robust and adaptive control strategies are essential for effectively responding to deviations from expected conditions^31^. As mentioned by^32^, the authors explored the uncertainty factors and modeling approaches related to integrating RESs and battery energy storage systems (BESS) into power grids. This study focused on power quality, battery degradation, and economic impact. It reviewed various uncertainty modeling approaches, including probabilistic, possibilistic, hybrid, GPR, robust optimization, and IGDT, highlighting their strengths and weaknesses. In reference^33^, the investigation focused on optimizing power flow in transmission and distribution systems, predicting short-term PV output, and developing coordination schemes between transmission system operators (TSOs) and distribution system operators (DSOs). The proposed approach enhanced prediction accuracy facilitated smoother integration of RESs and provided efficient optimal power flow solutions. However, it faced challenges related to system integration and weather dependencies. The results demonstrated accurate solar forecasting, optimized coordination between TSOs and DSOs, and improved system performance through a bi-level optimization approach. The study^34^ showed that grid computing enhanced smart grid performance by enabling real-time optimization, resource efficiency, and the integration of RESs. It supported demand response strategies and autonomous MG management while addressing resource allocation and security challenges. The approach improved electricity grid operation but requires reliable communication and efficient management. As noted by^35^, the authors developed a GPR model for reserve estimation in photovoltaic generators and simulated inertia for enhanced grid stabilization. The GPR model demonstrated superior accuracy and economic viability compared to the traditional deterministic model, improving grid stability. Besides, the PVG solution provided a cost-effective alternative to battery-based reserves. Another study^36^ focused on minimizing operational costs and pollutant emissions in MG dispatch by addressing the uncertainty in wind power forecasting through a probabilistic model and adaptive confidence interval (ACCI). It introduced the CSMOBA algorithm for better optimization performance and a two-step solution approach using CSMOBA and FTS. The results indicated that ACCI provided reliable forecasting, CSMOBA outperformed other algorithms, and the two-step approach efficiently identified optimal solutions. The research^37^ developed a combined data-driven and model-driven reliability evaluation framework for renewable energy distribution networks, with CWGAN used for scenario generation and mixed-integer linear programming applied for fault analysis. The advantages included improved accuracy, adaptability to RES uncertainty, and comprehensive fault analysis, while the disadvantages involved increased complexity and reliance on accurate data. The effectiveness of the framework in quantifying reliability was demonstrated, with it outperforming traditional methods, and validation was provided through simulation on a modified IEEE 123-bus system. Moreover, the reference^38^ proposed a stochastic simulation-optimization framework to address the uncertainties in RESs, utilizing statistics, stochastics, and copulas. This approach enhanced the design, risk assessment, and decision-making for hydro and wind power plants, with techno-economic performance enhanced. By capturing both internal and external uncertainties, it was shown to outperform deterministic models, offering a more realistic evaluation of RES investments.

Despite extensive research in MG management, significant gaps remain in addressing the critical factors that ensure robust and efficient system performance. Table 1 presents a comparative overview of the previous methods and the suggested method to show the originality of this research clearly.

**Table 1:** Consideration of key aspects in studies on microgrid optimization and Control.

Aspects	^32^	^33^	^34^	^35^	^36^	^37^	^38^	This study
Integration of RESs and ESS	✓	✓	✗	✗	✓	✗	✗	✓
Use of NLMPC	✗	✓	✗	✓	✗	✗	✗	✓
Considering forecast uncertainty by GPR	✓	✗	✗	✓	✗	✗	✗	✓
Multi objective optimization	✓	✗	✓	✓	✗	✓	✗	✓
Real time control strategy	✓	✗	✓	✗	✗	✓	✗	✓
EENS	✗	✗	✗	✗	✗	✓	✓	✓
Scalability and power-sharing	✗	✗	✗	✗	✗	✓	✓	✓
Uncertainty management	✓	✗	✗	✓	✗	✗	✗	✓
Operational cost and power quality	✓	✓	✗	✓	✗	✗	✗	✓

Previous studies exhibit limitations in several areas:

- Considering RESs and ESS: Although RESs have been considered in all previous works, the integration of energy storage system (ESS) has been insufficiently addressed. This paper bridges this gap by integrating both RESs and ESS to ensure enhanced system reliability and efficiency.
- Integration with AI Methods: Previous studies have overlooked the application of AI methods for optimizing MG performance. This study uniquely incorporates AI techniques, offering a more advanced and intelligent framework for real-time control.
- Operation Cost and Power Quality: While some studies have addressed operational costs, power quality—specifically voltage and frequency stability— has received limited attention. Cost reduction and power quality improvements are simultaneously emphasized in this research, filling this critical gap.
- EENS and Real-Time Performance: EENS has not been considered in previous works, leaving reliability-related issues unaddressed. Besides, while real-time performance has been considered in some studies, it still lacks comprehensive integration with technical and economic objectives. This paper proposes a new framework that effectively addresses both issues.
- Scalability Analysis and System Stability: Scalability and system stability indices have not been widely considered in previous works. By incorporating these factors, this study ensures the practicality of the framework for larger microgrid systems and facilitates the analysis of voltage and frequency stability.
- Uncertainty Management: While generation and consumption uncertainties were partially addressed in previous works, this research integrates advanced modeling techniques to better handle uncertainty, ensuring a more realistic representation of MG dynamics.

### Contribution

This study presents an advanced framework for optimizing MG operation using a data-driven NLMPC approach. The proposed method aims to improve the techno-economic efficiency of MGs by addressing uncertainties associated with RESs, load variability, and market price fluctuations. By integrating data-driven techniques with NLMPC, the framework enables real-time adaptation and boosts predictive accuracy, ensuring optimal power flow, minimizing operational costs, and improving system reliability. The contributions of this study are summarized as follows:

- This research introduces a novel NLMPC framework for hybrid MG control that easily integrates various energy sources, including utility power, wind turbines, microturbines, and battery energy storage. By utilizing the predictive capabilities of data-driven models, particularly GPR, the framework accurately predicts the dynamic behavior of DG units. This predictive capability is critical for addressing uncertainties associated with RES intermittency and load fluctuations, thereby enhancing the overall stability and resilience of the grid-connected MG.
- A key component of the proposed framework is the integration of Monte Carlo simulations to effectively model and address the uncertainties in grid-connected MG operations. This robustness of the NLMPC allows it to make well-informed control decisions under varying operating conditions. By using Monte Carlo simulations, the accuracy of system behavior predictions is improved, ensuring that the MG can adapt more reliably to changing circumstances.
- The suggested framework optimally allocates power flows and determines control points for DG units and loads while staying within system constraints. This approach enhances the accuracy of system behavior predictions, enabling the MG to adapt to more reliability to changing conditions.
- The data-driven NLMPC framework not only focuses on technical advancements but also takes into account the economic factor involved in MG management. This ensures that the solutions are both technically effective and economically feasible, promoting the sustainable and cost-effective implementation of hybrid MGs.

### Organization

This paper is structured as follows:the next section 2 provides a detailed explanation of the proposed framework and network configuration. The following section  3 describes the NLMPC method combined with data-driven techniques for MG management. Finally, the conclusion summarizes  5 the key findings and offering insights for future research aimed at optimizing microgrid performance through advanced data-driven control frameworks.

## Proposed framework

**Fig. 1:**
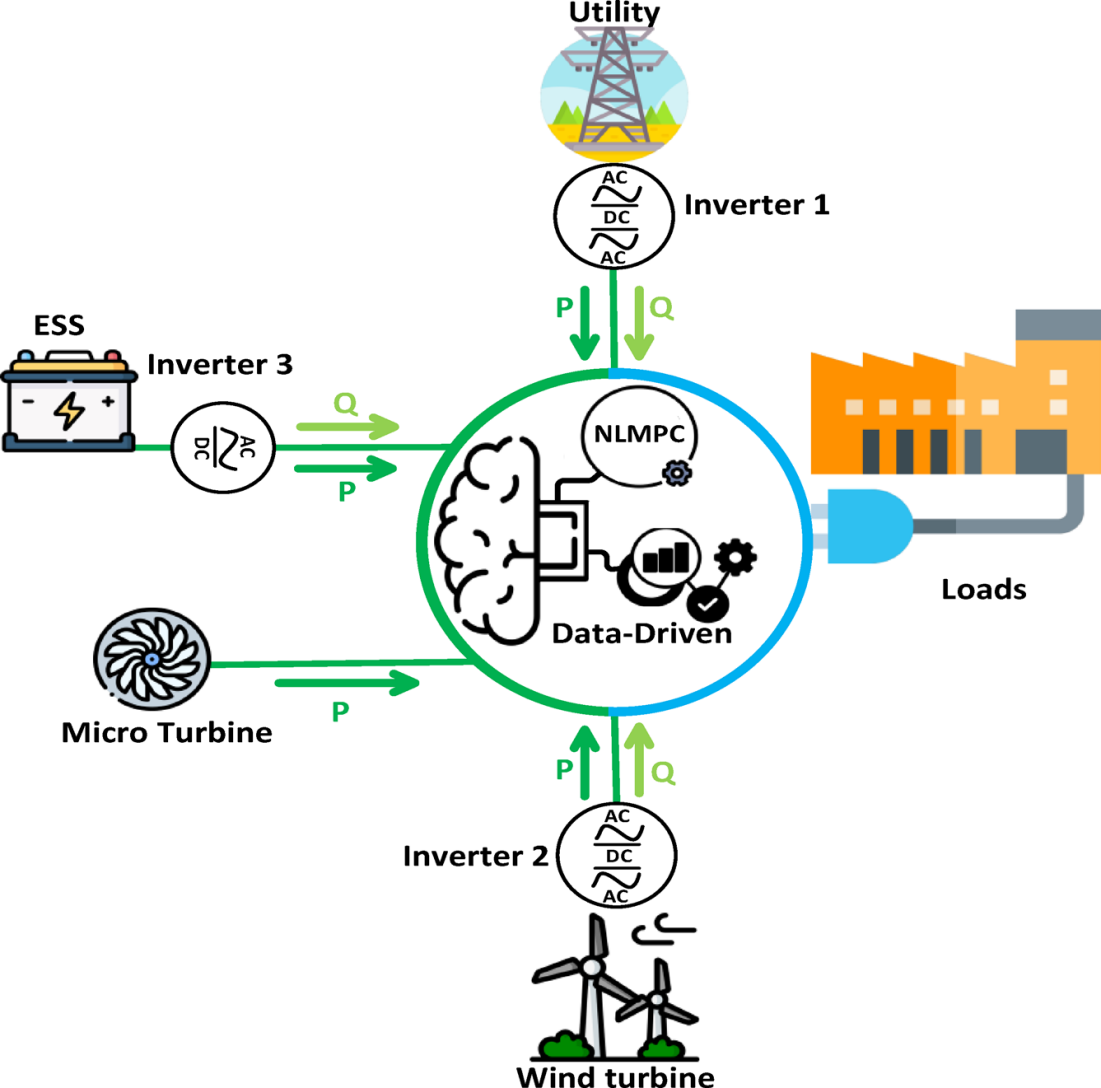
Conceptual model of the network and grid-connected MG.

Figure 1 depicts the data-driven NLMPC serving as the central controller within a grid-connected MG. The MG integrates utility system, wind turbines, a microturbine, and battery energy storage. The NLMPC is designed to optimize power flows and control setpoints across the DGs and local loads. The setup comprises three inverters:

- Inverter 1: Allocated to the utility, functioning as the slack bus (grid-forming bus).
- Inverter 2: Associated with wind turbines, operating as the PV bus (grid-coordinated bus).
- Inverter 3: connected to the battery storage, serving as the PQ bus (grid-following bus) during charging and transitioning to the PV bus during discharging.
- The microturbine is directly connected to the microgrid without an inverter.

In this setup, the NLMPC, utilizing data-driven approaches, manages the operation of the inverters and the microturbine. It ensures that power generation aligns with load demand while maintaining the required voltage and frequency levels, thereby supporting stable and efficient microgrid operations.

The data-driven NLMPC framework plays a pivotal role in managing microgrid operations by coordinating DG units and loads to attain optimal management. Integrating a data-driven approach with GPR, the NLMPC enables precise optimizations of power flow, minimizing energy losses while enhancing adaptability. By incorporating uncertainty models, the GPR-based NLMPC effectively predicts variable RES outputs and load fluctuations, enhancing system resilience and maintaining optimal performance despite dynamic changes in DG configurations or load variations. As the central control mechanism, the proposed strategy ensures voltage and frequency stability, maximizes energy efficiency, and guarantees a dependable power supply to demands. Energy storage systems, essential components of MGs, are typically modeled using state-space equations, with the state variable x(t) representing battery charge level^39^. These models form a robust foundation for predictive control, making state-space MPC ideal for managing the multi-variable dynamics of grid-connected MGs. MGs, characterized as multi-input, multiple-output (MIMO) systems, demand advanced control strategies to address their complex dynamics. The NLMPC framework determines a sequence of control inputs *u(t)* to optimize system outputs *y(t)* over a finite horizon, where outputs *y(t)* is often directly linked to state variables *x(t)*, and the output matrix *C* is an identity matrix. By using GPR-based predictions, the NLMPC accounts for the uncertainties in RESs and load demands. Its objective function balances economic and technical aspects, minimizing costs and energy losses while maintaining performance indices such as voltage and frequency regulation. Constraints on system dynamics, power balance, and operational limits are seamlessly integrated into the optimization process. At each sampling interval, the NLMPC predicts future states using current measurements and GPR-based models, ensuring smooth, reliable, and efficient MG operations. This advanced framework enables MGs to maintain resilience, adaptability, and high performance in dynamic and uncertain conditions. Hence, Eq. 1 shows the objective function that encompasses both economic and technical considerations within the data-driven NLMPC approach, while incorporating GPR to address system uncertainties.

1 $${\mathbf{minJ=}}\sum\limits_{{{\mathbf{K=0}}}}^{{\mathbf{N}}} {{\mathbf{(y(t+k)-}}{{\mathbf{y}}_{{\mathbf{ref}}}}{\mathbf{(t+k)||}}_{{\mathbf{Q}}}^{{\mathbf{2}}}{\mathbf{+u(t+k)-}}{{\mathbf{u}}_{{\mathbf{prev}}}}{\mathbf{(t+k)||}}_{{\mathbf{R}}}^{{\mathbf{2}}}{\mathbf{)}}}$$

Where, the cost function balancing technical and economic objectives is shown by ${\mathbf{J}}$. the desired reference output,${{\mathbf{y}}_{{\mathbf{ref}}}}$, encompasses both economic and technical indexes. The control input ${\mathbf{u}}$ serves as a signal to regulate *Id* and *Iq*. While ${{\mathbf{u}}_{{\mathbf{prev}}}}$ is to penalize deviations in the predicted control inputs across the prediction horizon.${\mathbf{R}}$and${\mathbf{Q}}$illustrate the weighting matrices for tracking performance and input changes.

## NLMPC with Data-Driven techniques for microgrid management

Data-driven methods rely on system data to learn behavior, eliminating the need for precise physical models. In NLMPC, these methods are employed to model system dynamics and predict the future responses of DGs. In the data-driven approach, the traditional dynamic models, typically expressed as:

2 $$x(t+1)=\Im (x(t),u(t))$$

**Fig. 2:**
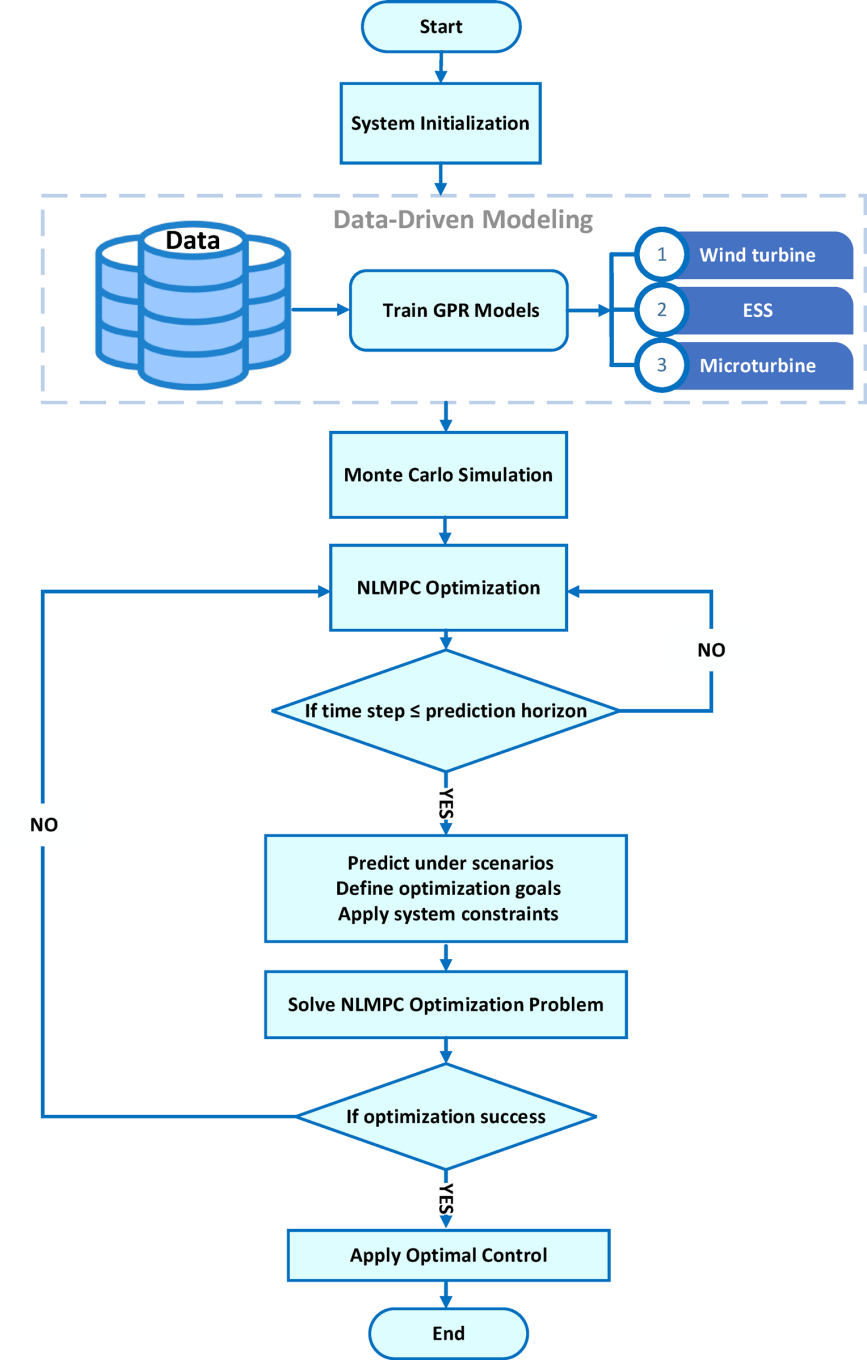
Flowchart of Data-driven NLMPC for optimized MGs under uncertainties.

Where$x(t)$ is the state vector at time *t*, $u(t)$is the control input and $\Im$donates the true system dynamic. In the data-driven approach, this model is replaced with a learning-based representation such as:

3 $$x(t+1)={\hat {\Im }_{GPR}}(x(t),u(t))$$

where, ${\hat {\Im }_{GPR}}$ represents the system dynamics modeled using GPR. GPR leverages system data to capture complex relationships and uncertainties in the dynamics, allowing for accurate predictions of how the system responds to input changes and environmental factors, such as load variations and renewable energy fluctuations. This enhances the precision of future predictions and ensures more robust control in uncertain and dynamic conditions. Additionally, this framework effectively addresses uncertainties such as load fluctuations, variations in wind speed, and resource constraints. By integrating these uncertainties into the data and utilizing Monte Carlo simulation techniques, the system achieves substantially enhanced predictive accuracy and reliability. As a result, the data-driven NLMPC emerges as a powerful and reliable tool for managing MG operations in dynamic and uncertain environments. Figure 2 shows the Flowchart of Data-driven NLMPC for optimized MGs under uncertainties. To clarify, Algorithm 1 provides a step-by-step illustration of the data-driven NLMPC approach for MGs under uncertain conditions.

**Figure:**
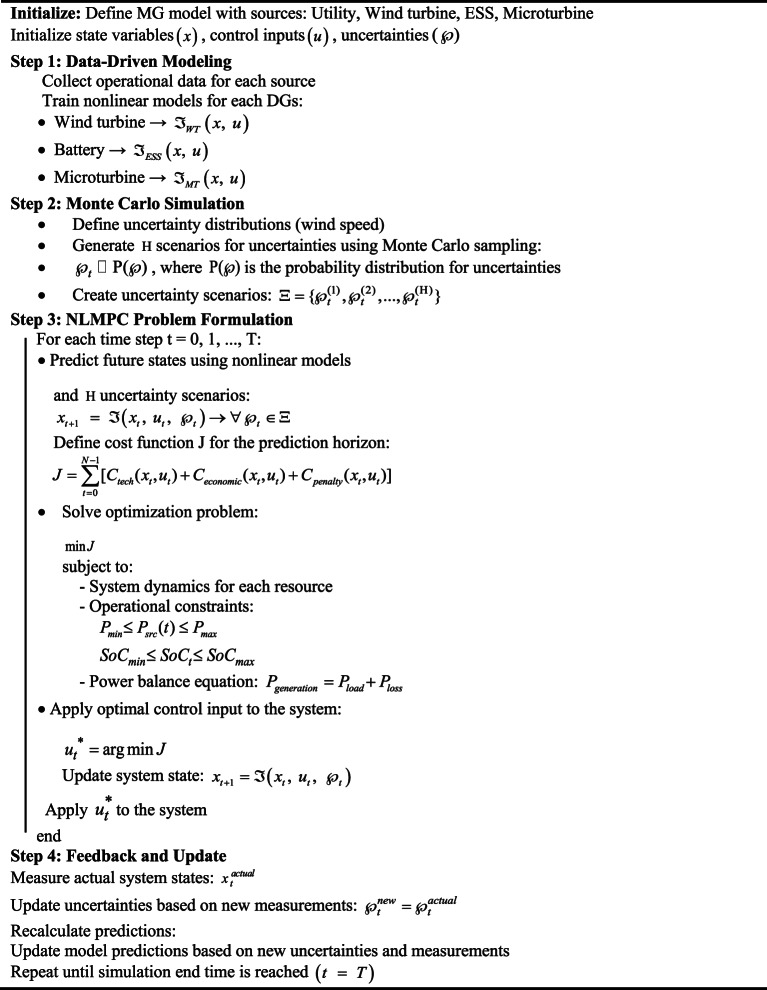
**Algorithm1:** Data-driven NLMPC of MGs under uncertainties

### Gaussian process regression (GPR)

The primary strength of GPR lies in its ability to make predictions with inherent uncertainty estimation, providing confidence intervals for its outputs. This feature is particularly valuable for MG optimization, where real-time decisions often need to account for dynamic and uncertain conditions such as fluctuations in RESs, varying loads, and resource constraints. By incorporating these probabilistic insights, GPR enables the data-driven NLMPC framework to adapt and respond more effectively to real-world complexities. In the proposed Algorithm 2, GPR serves as the core modeling technique for representing the MG dynamics. Instead of relying solely on deterministic physical models, the algorithm uses GPR to learn the relationships between the state variables $x(t)$, control inputs $u(t)$, and the predicted system state $x(t+1)$. This probabilistic modeling approach offers a more precise and reliable representation of MG behavior under conditions of uncertainty.

**Figure:**
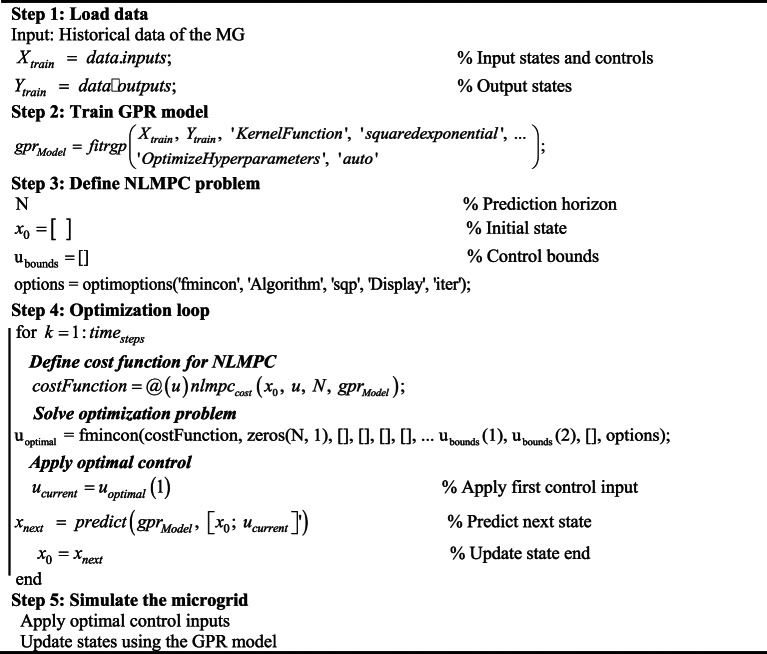
**Algorithm 2:** Data-driven NLMPC using GPR for real-time MG optimization

The uncertainty quantification capability of GPR generates confidence intervals for predictions, which enable the NLMPC algorithm to assess control strategies considering the risks associated with uncertain outcomes. Its data efficiency makes it an excellent choice for situations when long historical data may not be available, and its adaptability allows the model to dynamically adjust to real-time changes in the microgrid, such as load variations or renewable energy variability. By enhancing predictive accuracy and thus supporting informed decision-making, the integration of GPR transforms the NLMPC framework into a robust solution for microgrid optimization. The algorithm leverages the ability of GPR to handle nonlinearity and uncertainty, optimizing control inputs *u(t)* to achieve objectives, such as minimizing energy losses, ensuring voltage and frequency stability, and maintaining a reliable power supply.

Moreover, to justify the selection of GPR as the predictive model at the suggested data-driven NLMPC framework, a comparative assessment was conducted against other AI/ML-based approaches that have recently gained increasing attention in the literature, as indicated in Table 2.

**Table 2:** Comparative evaluation of GPR with other AI/ML models for microgrid Optimization.

Model	Feature selection support	Prediction accuracy (RMSE ↓)	Interpretability	Uncertainty quantification	Notes
GPR (suggested framework)	Moderate (embedded in kernel functions)	0.13 (validated on RES output forecast)	High (probabilistic and analytical)	Yes (confidence intervals)	Ideal for data-scarce environments with uncertainty
Random Forest ^40^	Strong (feature importance ranking)	0.16	Moderate (tree-based interpretability)	No	Requires tuning, less effective for extrapolation
LSTM ^42^	Weak (requires external FS method)	0.15	Low (black-box model)	No	Needs large datasets; not optimal for short-term real-time control
XGBoost ^42^	Strong (gain and SHAP values)	0.14	Moderate (post-hoc explain ability)	No	Fast training, but lacks native uncertainty estimates

According to Table 2, a comparative analysis was made, and the results show that while methods such as decision trees, support vector machines, and ensemble models exhibit strong performance in certain situations, GPR is beneficial since it enables the quantification of predictive uncertainty. This probabilistic nature is critical in MG optimization problems where decision-making needs to account for unstable and uncertain operating conditions. By integrating GPR into the suggested NLMPC framework, the system gains enhanced adaptability to real-time behavior while maintaining robustness and interpretability.

## Problem formulation

This part presents the objectives and constraints of the suggested strategy. The objective functions focus on major technical aspects, such as power loss (PL), operating cost, frequency and voltage stability, voltage deviation (VD), expected energy not supplied (EENS), load ability limited (LAL), and management of storage devices. The subsections present the mathematical formulations and limitations related to all DGs. In real-time applications, the method is evaluated based on its ability to quickly estimate stability margins without post-fault data while keeping computational costs low. Nevertheless, recalculating the operating point of the network is difficult due to configuration modifications caused by disruptions. To address these challenges, Network-Preserving Models (NPMs) offer a significant advantage over traditional network-reduction models. Initially, network-reduction models were used, however, they had limitations in accurately representing the system structure, especially in dynamic situations. In contrast, NPMs preserve the network topology, providing a more practical depiction of electric power system components, comprising dynamic demand behavior and detailed generator models. This approach leads to the development of a structure-preserving energy function that offers a more accurate approximation of the actual behavior of the system. Moreover, NPMs offer significant computational advantages. By utilizing sparse matrix techniques, nonlinear algebraic equations inherent in the model can be solved efficiently. This enables more accurate and practical real-time stability analysis, ensuring better performance compared to the earlier network-reduction models.

### Inverter modeling for microgrid

Figure 2 presents the inverter model with an LC filter as part of the data-driven NLMPC, which uses a nonlinear control strategy to optimize system behavior. The approach uses a mathematical model of the grid-connected MG to predict future performance, incorporating nonlinear dynamics from components like inverters, microturbines, and energy storage^43^. This model captures key system interactions and enhances control decisions by considering factors such as renewable energy, battery charge, load forecasts, and system constraints, With the aim of reducing costs and emissions while improving reliability. The terminal voltage and phase angle of the inverter, after passing through the LC filter, are represented as${\mathbf{U}}\angle {\mathbf{\theta }}$. This model allows the system dynamics to be captured using only the terminal states of the inverter (angle, frequency, and voltage) along with line currents, without requiring the internal states of the inverter. It is based on a 5th-order electromagnetic framework that utilizes the dq reference frame. Equation (4) to (6) describe the inverter-related states, while Eqs. (7) and (8) represent the line-related states.

**Fig. 3:**
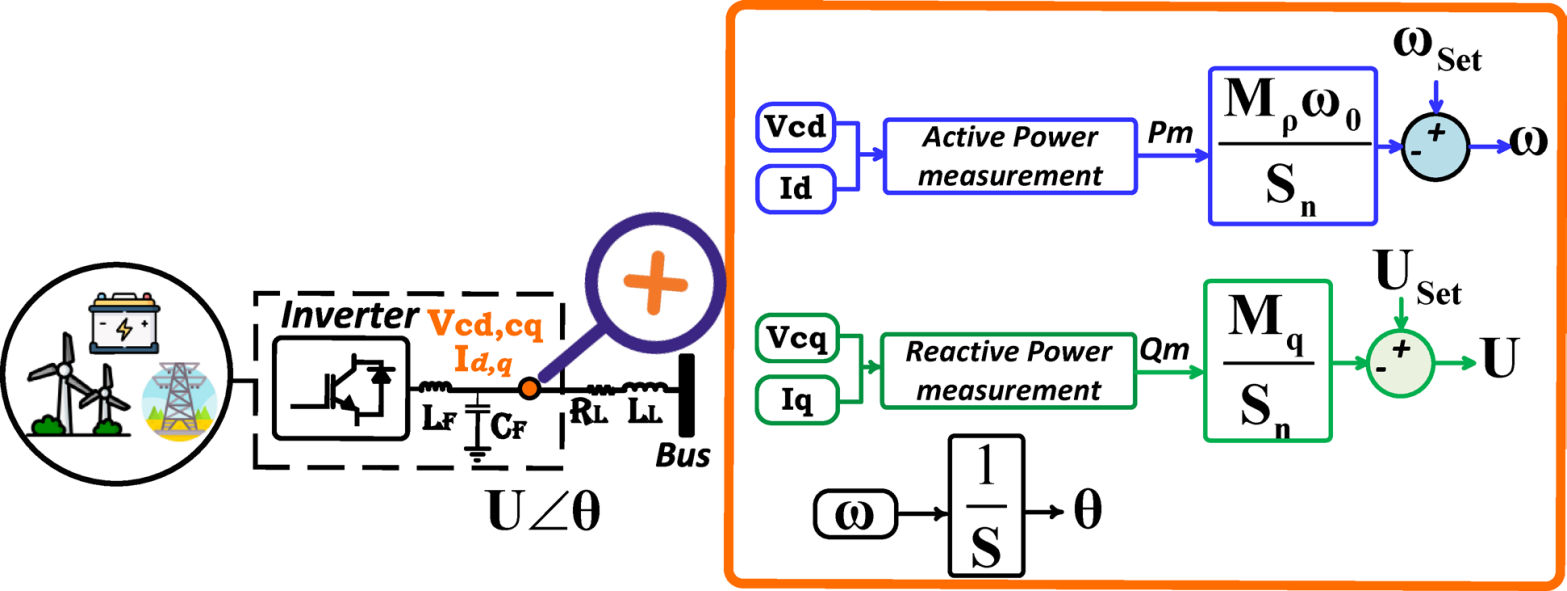
Inverter model with LC filter in the data-driven NLMPC framework.

Equations for inverter Dynamics:

4 $${\mathbf{\theta =\omega -}}{{\mathbf{\omega }}_{\mathbf{0}}}$$

5 $${\mathbf{\varsigma \omega ^{\prime}=}}{{\mathbf{\omega }}_{{\mathbf{Set}}}}{\mathbf{-\omega -}}\frac{{{{\mathbf{M}}_{\mathbf{p}}}{{\mathbf{\omega }}_{\mathbf{0}}}}}{{{{\mathbf{S}}_{\mathbf{n}}}}}{{\mathbf{P}}_{\mathbf{m}}}$$

6 $${\mathbf{\varsigma }}\mathop {\mathbf{U}}\limits^{{\mathbf{ \bullet }}} {\mathbf{=}}{{\mathbf{U}}_{{\mathbf{Set}}}}{\mathbf{-U-}}\frac{{{{\mathbf{M}}_{\mathbf{q}}}}}{{{{\mathbf{S}}_{\mathbf{n}}}}}{{\mathbf{Q}}_{\mathbf{m}}}$$

Equations for line-related states:

7 $$l{{\mathbf{{\rm I}^{\prime}}}_{\mathbf{d}}}{\mathbf{=}}{{\mathbf{U}}_{{\mathbf{cos\theta }}}}{\mathbf{-}}{{\mathbf{U}}_{\mathbf{0}}}{\mathbf{-R}}{{\mathbf{{\rm I}}}_{\mathbf{d}}}{\mathbf{+}}{{\mathbf{\omega }}_{\mathbf{0}}}l{{\mathbf{{\rm I}}}_{\mathbf{d}}}$$

8 $$l{{\mathbf{{\rm I}^{\prime}}}_{\mathbf{d}}}{\mathbf{=}}{{\mathbf{U}}_{{\mathbf{sin\theta }}}}{\mathbf{-}}{{\mathbf{U}}_{\mathbf{0}}}{\mathbf{-R}}{{\mathbf{{\rm I}}}_{\mathbf{d}}}{\mathbf{+}}{{\mathbf{\omega }}_{\mathbf{0}}}l{{\mathbf{{\rm I}}}_{\mathbf{d}}}$$

In this case, the variable ${\mathbf{U}}$and ${\mathbf{\theta }}$ the effective terminal voltage and phase angle of the inverter, respectively, after passing through the LC filters. The variable ${\mathbf{\omega }}$ indicates the frequency of the inverter while ${{\mathbf{I}}_{\mathbf{d}}}$ and ${{\mathbf{I}}_{\mathbf{q}}}$ the dq-frame components of the inverter output current. Equations (5) and (6) represent the behavior of the terminal voltage and frequency, respectively.

These equations account for the effect of low-pass filters in the power control system of the inverter, with a bandwidth given by:

9 $${{\mathbf{\omega }}_{{\mathbf{filter}}}}{\mathbf{=\varsigma -1}}$$

The parameters ${{\mathbf{M}}_{\mathbf{p}}}$ and ${{\mathbf{M}}_{\mathbf{q}}}$ are the frequency and voltage droop gains, respectively. Additionally, ${{\mathbf{S}}_{\mathbf{n}}}$represents the rated capacity of the inverter, while ${{\mathbf{\omega }}_{{\mathbf{Set}}}}$ and ${{\mathbf{U}}_{{\mathbf{Set}}}}$ the predefined set points for frequency and voltage controllers, respectively, which serve as inputs to the inverter. The variables${L_T}={L_F}+{L_L}$(mH) and ${R_T}={R_F}+{R_L}$(mΩ) represent the total inductance and resistance of the connection, respectively, as measured at the inverter terminal. The expressions for the measured active power and reactive power are given by:

10 $${{\mathbf{P}}_{\mathbf{m}}}{\mathbf{=}}\frac{{\mathbf{3}}}{{\mathbf{2}}}{\mathbf{U}}{{\mathbf{I}}_{\mathbf{d}}}$$

11 $${{\mathbf{Q}}_{\mathbf{m}}}{\mathbf{=-}}\frac{{\mathbf{3}}}{{\mathbf{2}}}{\mathbf{U}}{{\mathbf{I}}_{\mathbf{q}}}$$

### Modeling of wind turbine

The power output of a wind turbine is a nonlinear function of wind speed, rotor speed, and power coefficient as shown in:

12 $${P_{WT}}(t)=\frac{1}{2}.\Upsilon .{\rm A}.{C_P}(\ell ,\beta ).V_{{Wind}}^{3}(t)$$

$\Upsilon$is the air density, ${\rm A}$is the swept area of the wind turbine rotor.${C_P}(\ell ,\beta )$ is the power coefficient as a function of tip-speed ratio ($\ell$) and pitch angle ($\beta$). ${V_{Wind}}(t)$is the wind speed at time *t.*

However, when modeling with GPR, several factors such as wind speed, weather conditions, and turbine performance are considered. Therefore, the power output of the wind turbine is represented by the following equation:

13 $${P_{WT}}(t)={\Im _{WT}}({V_{Wind}}(t),\wp )$$

Where, ${\Im _{WT}}$is the GPR model predicts the output power based on the speed ${V_{Wind}}(t)$ and the uncertainties parameter is$\wp$, which include the weather.

### Modeling of ESS

The energy storage system can be modeled by its state of charge (SoC), efficiency, and dynamic behavior. The power output or input is controlled by the charging or discharging process:

14 $$SoC(t)=SoC(t - 1)+\frac{{{I_{ESS}}(t).\Delta t}}{{{C_{ESS}}}}$$

Where, $SoC(t)$ is state of charge at time *t*. Also, ${I_{ESS}}(t)$ is the current (positive for discharge, negative for charge) at time *t*, ${V_{ESS}}$ is the voltage of the ESS.${C_{ESS}}$is the capacity of the energy storage system.$\Delta t$is the time step.

15 $$\begin{gathered} {P_{ESS - Ch\arg e}}(t)={\eta _{Ch\arg e}}.{I_{ESS}}(t).{V_{ESS}} \hfill \\ {P_{ESS - Disch\arg e}}(t)={\eta _{Disch\arg e}}.{I_{ESS}}(t).{V_{ESS}} \hfill \\ \end{gathered}$$

The ${P_{ESS - Ch\arg e}}(t)$is the power during the charging, while ${P_{ESS - Disch\arg e}}(t)$ is the power during the discharging.${\eta _{Ch\arg e}}$is the charging efficiency while ${\eta _{Disch\arg e}}$is the efficiency during discharge. In the case of energy storage, GPR can predict the charging and discharging behavior of the battery, simulating its operation under various conditions:

16 $$SoC(t)={\Im _{SoC}}({I_{ESS}}(t),\wp )$$

Where,${\Im _{SoC}}({I_{ESS}}(t),\wp )$is a GPR model that uses operational data to predict the battery state of charge based on current input (${I_{ESS}}(t)$) and other influencing parameters are shown by $\wp$. Power input/output of ESS with GPR follows below equation:

17 $${P_{ESS}}(t)={\Im _{ESS}}({I_{ESS}}(t),\wp )$$

${\Im _{ESS}}({I_{ESS}}(t),\wp )$is a GPR model that predicts the input/output power of the ESS based on real-world data instead of classical equations.

### Modeling of microturbine

The microturbine model is based on the thermodynamic process and its power generation capability, which depends on fuel consumption, turbine efficiency, and load, as shown in Eq. (18):

18 $${P_{MT}} (t)= {\eta {MT}}. {\lambda{Fuel}} (t).\Delta {\hbar _{Fuel}} $$

${\eta _{MT}}$is the efficiency of the microturbine, ${\mathchar26\mkern-10mu\lambda _{Fuel}}(t)$is the mass flow rate of the fuel at time *t*. $\Delta {\hbar _{Fuel}}$is the enthalpy difference of the fuel. The power output of the microturbine, when modeled using GPR, is described by the following equation. GPR enables accurate prediction of microturbine behavior using input data such as fuel consumption, pressure, and rotational speed, effectively capturing the system nonlinear dynamics and performance characteristics:

19 $${P_{MT}}(t)={\Im _{MT}}({\\\lambda _{Fuel}}(t),\wp )$$

Where, $${\Im _{MT}}({\\\lambda _{Fuel}}(t),\wp )$$ is a GPR model that predicts the power output of the microturbine with fuel consumption ($${\\\lambda _{Fuel}}(t)$$) and environmental conditions ($\wp$).

### Modeling of cost

The cost models for wind turbines, energy storage systems, microturbines, and the main grid are formulated as follows: the wind turbine cost is presented in Eq. (20), and the energy storage system cost is represented in Eq. (21).

20 $$\operatorname{Cos} {t^{WindTurbine}}(t)=\frac{{\operatorname{Cos} t_{{capital}}^{{wind}} \times \rho _{{capital}}^{{wind}} \times Gr}}{{{T_{life}} \times 365 \times 24 \times C{F^{wind}}}}+\operatorname{Cos} t_{{O\& M}}^{{wind}} \times \rho (t)$$

21 $$\operatorname{Cos} {t^{ESS}}(t)=\frac{{\operatorname{Cos} t_{{capital}}^{{ESS}} \times \rho _{{capital}}^{{ESS}} \times Gr}}{{{T_{life}} \times 365 \times 24 \times C{F^{ESS}}}}+\operatorname{Cos} t_{{O\& M}}^{{ESS}} \times \left| {{\rho ^{ESS}}(t)} \right| \pm {{\rm B}^{TOU}}(t) \times {\rho ^{ESS}}(t)$$

The cost of wind turbines and energy storage devices is represented by a linear equation, where, $\operatorname{Cos} t_{{capital}}^{{ESS}}$ is the capital cost of ESS, and $\rho _{{capital}}^{{ESS}}$is the investment cost per unit of capacity for ESS. The annual interest rate is $Gr$. The variable ${T_{life}}$shows the lifetime of ESS. $C{F^{ESS}}$is the capacity factor of ESS. ${\rho ^{ESS}}(t)$is the charging or discharging capacity of ESS. ${{\rm B}^{TOU}}$ represents time-of-use pricing adjustments.

Variable costs include operating and maintenance expenses:

22 $$\left. \begin{gathered} \operatorname{Cos} {t^\tau }=\operatorname{Cos} t_{{O\& M}}^{\tau }(t)+{\kappa ^\tau } \times \operatorname{Cos} t_{{EM}}^{\tau }(t) \hfill \\ \operatorname{Cos} t_{{O\& M}}^{\tau }(t)=\alpha \times \rho _{\tau }^{2}(t)+\beta \times {\rho _\tau }(t)+\gamma \hfill \\ \operatorname{Cos} t_{{EM}}^{\tau }(t)=(C_{{C{O_2}}}^{\tau }+C_{{S{O_2}}}^{\tau }+C_{{N{O_x}}}^{\tau }) \times {\rho _\tau }(t) \hfill \\ \end{gathered} \right\}\forall \tau \in Microturbine,Grid$$

For the microturbine and main grid, the total operating cost consists of two components: emissions cost ($\operatorname{Cos} {t_{EM}}$) and operation and maintenance cost ($\operatorname{Cos} {t_{O\& M}}$). Emission cost is defined as the sum of the pollutant gases, including carbon dioxide $C{O_2}$, sulfur dioxide $S{O_2}$, and nitrogen oxides $N{O_x}$, which are the most harmful pollutants in the power grid. The fuel cost of the combined cycle gas turbines is represented by a quadratic function. The generation cost is the function of actual power and cost coefficients ($\alpha$,$\beta$,$\gamma$). The price penalty coefficient is (${\kappa ^\tau }$), as presented in Eq. (23):

23 $${\kappa ^\tau }={\left. {\frac{{\operatorname{Cos} t_{{O\& M}}^{\tau }(t)}}{{\operatorname{Cos} t_{{EM}}^{\tau }(t)}}} \right|_{\rho _{{\hbox{max} }}^{\tau }}}$$

### Modeling of monte carlo

Monte Carlo simulation is an effective method for modeling uncertainties in wind turbine performance, especially in power generation, where uncertainties arise from variations in wind speed, air density, and turbine efficiency. By performing a large number of iterations, the simulation generates a distribution of possible outcomes, allowing for a thorough assessment of the uncertainty in the model. In this approach, uncertain parameters such as wind speed and air density are treated as random variables with known distributions-in this case, normal distribution. Each iteration involves sampling these random variables, with wind speed${\nu _i}$, and air density${p_i}$ drawn from normal distributions $N({\mu _\nu },{\sigma _\nu })$and$N({\mu _p},{\sigma _p})$respectively, and other parameters like tip-speed ratio${\lambda _i}$, pitch angle${{\rm E}_i}$, and efficiency${\eta _i}$sampled from appropriate distributions. The power output for each iteration is then calculated using the power output function ${P_i}=f({\nu _i},{p_i},{\lambda _i},{{\rm E}_i},{\eta _i})$.After performing *N* iterations (in this case 10000), the resulting power outputs${P_i}$are analyzed statistically. The mean power output is calculated:

24 $${\mu _{Power}}=\frac{1}{N}\sum\limits_{{i=1}}^{N} {{P_i}}$$

Moreover, the standard deviation calculated as:

25 $${\sigma _{Power}}=\sqrt {\frac{1}{N}\sum\limits_{{i=1}}^{N} {({P_i}} - {\mu _p}{)^2}}$$

Additionally, the 95% confidence interval ($CI$) for the power output can be deduced from the 2.5th and the 97.5th percentiles of the power output distribution, denoted as:

26 $$C{I_{95\% }}=[{P_{2.5th}},{P_{97.5th}}]$$

To provide further clarity, Algorithm 3 describes the specific steps considered in this study, including the exact number of iterations and distributions used for each parameter.

**Figure:**
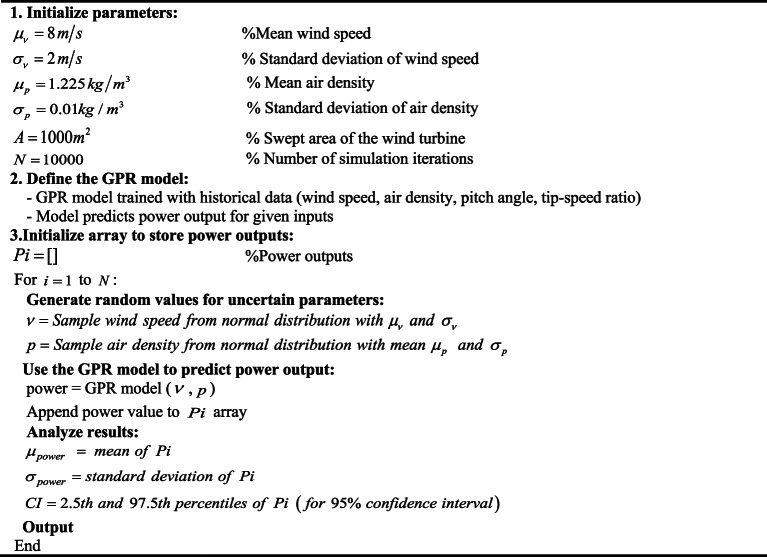
**Algorithm 3:** Monte Carlo Simulation for wind turbine power output uncertainty

### Dynamic control models for system optimization

This study investigates a grid-connected MG consisting of three inverters, with the microturbine connected directly to the grid. As mentioned in Eq. (4) to (8), each inverter has five equations. In this section, the equations related to each inverter are explained in detail to show how many equations each inverter includes based on the considered setup. Equation (27) to (30) introduce the equations governing the inverters. Each equation describes how the state variables are updated at each time step, based on the system’s previous state and the applied control inputs.

The differential equations governing the inverter of the main grid are given by Eq. (26). In this regard, the state vector${[{{\rm X}_1}({\rm Z}),...,{{\rm X}_5}({\rm Z})]^T}$, with items${[{\theta _1}({\rm Z}),{\omega _1}({\rm Z}),{u_1}({\rm Z}),{I_{d1}}({\rm Z}),{I_{q1}}({\rm Z})]^T}$, reflects the dynamic variables of inverter 1. The control inputs are captured in the vector${[{u_1}({\rm Z}),{u_2}({\rm Z})]^T}$, which are associated with inverter 1 and manage its operation, with the elements ${[{\omega _{Set1}}({\rm Z}),{u_{Set1}}({\rm Z})]^T}$ serving as control signals:

27 $$\begin{gathered} {{\rm X}_1}({\rm Z}+1)={\Im _1}({\rm X}({\rm Z})) \hfill \\ {{\rm X}_2}({\rm Z}+1)={\Im _2}({\rm X}({\rm Z}),u({\rm Z})) \hfill \\ {{\rm X}_3}({\rm Z}+1)={\Im _3}({\rm X}({\rm Z}),u({\rm Z})) \hfill \\ {{\rm X}_4}({\rm Z}+1)={\Im _4}({\rm X}({\rm Z})) \hfill \\ {{\rm X}_5}({\rm Z}+1)={\Im _5}({\rm X}({\rm Z})) \hfill \\ \end{gathered}$$

Equation (28) describes the differential equations that govern the operation of the wind turbine inverter. The state vector${[{{\rm X}_6}({\rm Z}),...,{{\rm X}_{10}}({\rm Z})]^T}$, with element ${[{\theta _2}({\rm Z}),{\omega _2}({\rm Z}),{u_2}({\rm Z}),{I_{d2}}({\rm Z}),{I_{q2}}({\rm Z})]^T}$, represents the dynamic variables of inverter 2. The control inputs for inverter 2 are represented by the vector${[{u_3}({\rm Z}),{u_4}({\rm Z})]^T}$, which includes the control signals to manage its operation:

28 $$\begin{gathered} {{\rm X}_6}({\rm Z}+1)={\Im _6}({\rm X}({\rm Z})) \hfill \\ {{\rm X}_7}({\rm Z}+1)={\Im _7}({\rm X}({\rm Z}),u({\rm Z})) \hfill \\ {{\rm X}_8}({\rm Z}+1)={\Im _8}({\rm X}({\rm Z}),u({\rm Z})) \hfill \\ {{\rm X}_9}({\rm Z}+1)={\Im _9}({\rm X}({\rm Z})) \hfill \\ {{\rm X}_{10}}({\rm Z}+1)={\Im _{10}}({\rm X}({\rm Z})) \hfill \\ \end{gathered}$$

The differential equations for the microturbine are defined by Eq. (29). The microturbine is directly connected to the network and can only provide active power to maintain greater control degrees of freedom within the system. Therefore, there are three differential equations for the microturbine. The state vector is represented by ${[{{\rm X}_{11}}({\rm Z}),{{\rm X}_{12}}({\rm Z}),{{\rm X}_{13}}({\rm Z})]^T}$. The control input for microturbine is${[{u_5}({\rm Z})]^T}$. As a result, three differential equations are considered for microturbine in this configuration:

29 $$\begin{gathered} {{\rm X}_{11}}({\rm Z}+1)={\Im _{11}}({\rm X}({\rm Z})) \hfill \\ {{\rm X}_{12}}({\rm Z}+1)={\Im _{12}}({\rm X}({\rm Z}),u({\rm Z})) \hfill \\ {{\rm X}_{13}}({\rm Z}+1)={\Im _{13}}({\rm X}({\rm Z})) \hfill \\ \end{gathered}$$

The differential equations for the ESS inverter are defined by Eq. (30). In addition to Eq. (4) to (8), this inverter has two additional differential equations that account for the SoC and energy within the ESS. The state vector${[{{\rm X}_{14}}({\rm Z}),...,{{\rm X}_{20}}({\rm Z})]^T}$, with elements ${[{\theta _3}({\rm Z}),{\omega _3}({\rm Z}),{u_3}({\rm Z}),{I_{d3}}({\rm Z}),{I_{q3}}({\rm Z})]^T}$, represents the dynamic variables of inverter 3. The control inputs for inverter ESS are${[{u_6}({\rm Z}),{u_7}({\rm Z})]^T}$, corresponding to control signals${[{\omega _{Set3}}({\rm Z}),{u_{Set3}}({\rm Z})]^T}$to manage its operation:

30 $$\begin{gathered} {{\rm X}_{14}}({\rm Z}+1)={\Im _{14}}({\rm X}({\rm Z})) \hfill \\ {{\rm X}_{15}}({\rm Z}+1)={\Im _{15}}({\rm X}({\rm Z}),u({\rm Z})) \hfill \\ {{\rm X}_{16}}({\rm Z}+1)={\Im _{16}}({\rm X}({\rm Z}),u({\rm Z})) \hfill \\ {{\rm X}_{17}}({\rm Z}+1)={\Im _{17}}({\rm X}({\rm Z})) \hfill \\ {{\rm X}_{18}}({\rm Z}+1)={\Im _{18}}({\rm X}({\rm Z})) \hfill \\ {{\rm X}_{19}}({\rm Z}+1)={\Im _{19}}({\rm X}({\rm Z})) \hfill \\ {{\rm X}_{20}}({\rm Z}+1)={\Im _{20}}({\rm X}({\rm Z})) \hfill \\ \end{gathered}$$

## Simulation and results

**Fig. 4:**
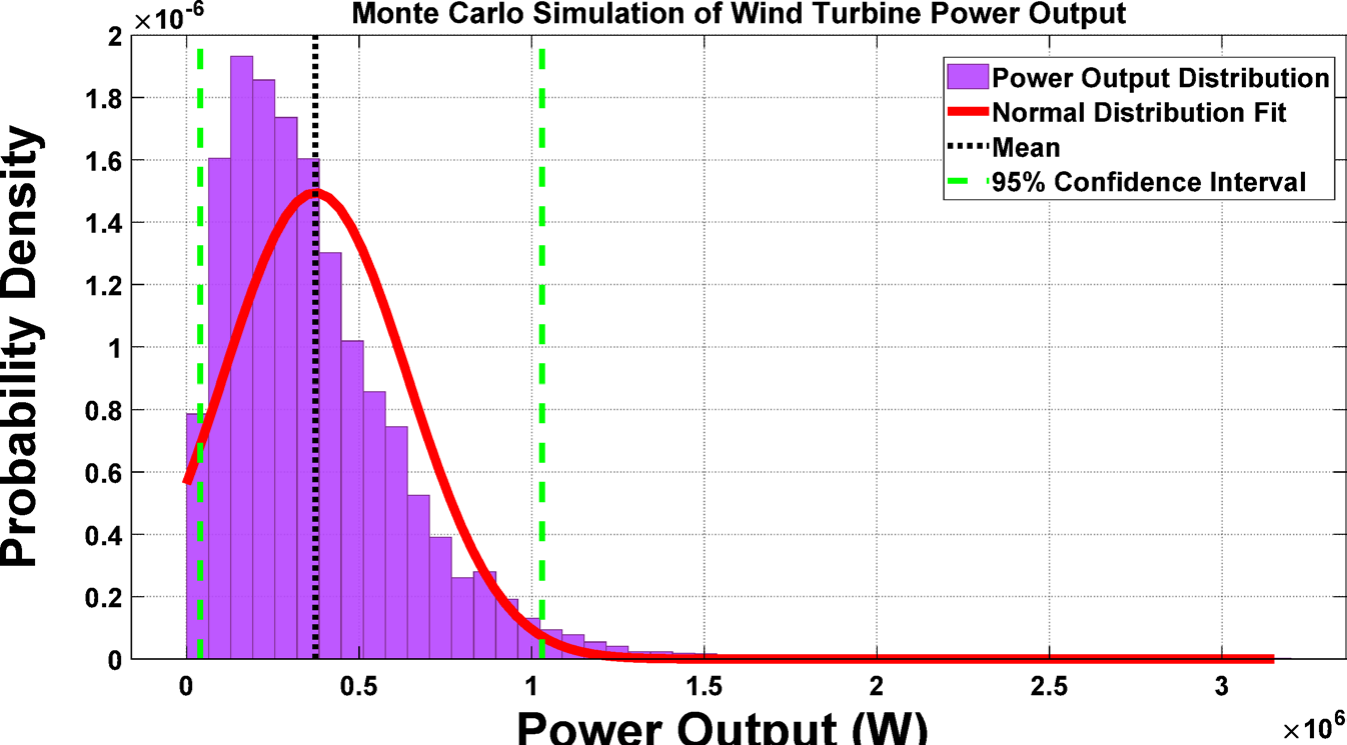
Wind speed variations in the grid-connected MG based on Monte Carlo simulation to model uncertainties and assess their impact on power generation.

Figure 4 illustrates the uncertainties in wind speed, air density, and turbine efficiency. These uncertainties are simulated by Monte Carlo simulation to effectively reflect the fluctuation and provide a more accurate representation of these parameters.

**Fig. 5:**
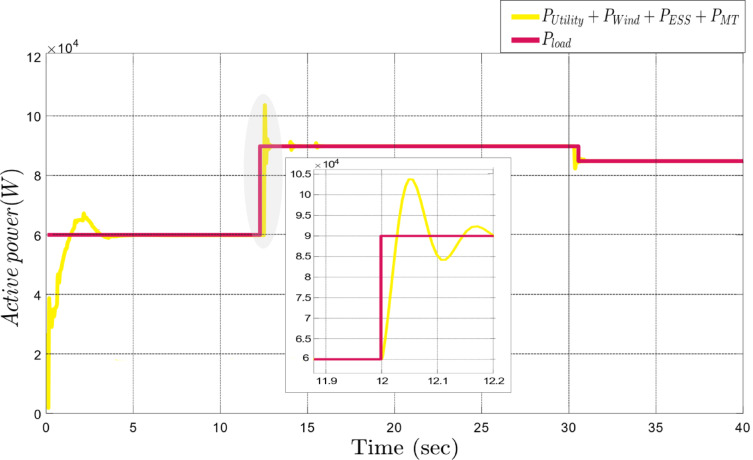
Dynamic response of the proposed framework to sudden active power changes.

In this study, sudden changes in active and reactive power are employed to evaluate the performance of the proposed framework. Figure 5 depicts the operation when, at the 12th second, the active power suddenly increases from 60 kW to 90 kW. As a response to this jump, the proposed framework automatically reacts and performs the optimal power sharing among DGs, which is effectively captured in the zoomed portion of this figure. At the 31 st second, to further ensure the reliability of the framework, a sudden decrease in active power from 90 to 85 kW was simulated. This reduction was applied to assess the ability of the system to manage power sharing even under abrupt decreases in active power demand. As shown in Fig. 5, the proposed framework also responds promptly to this decrease, while its performance remains stable throughout.

**Fig. 6:**
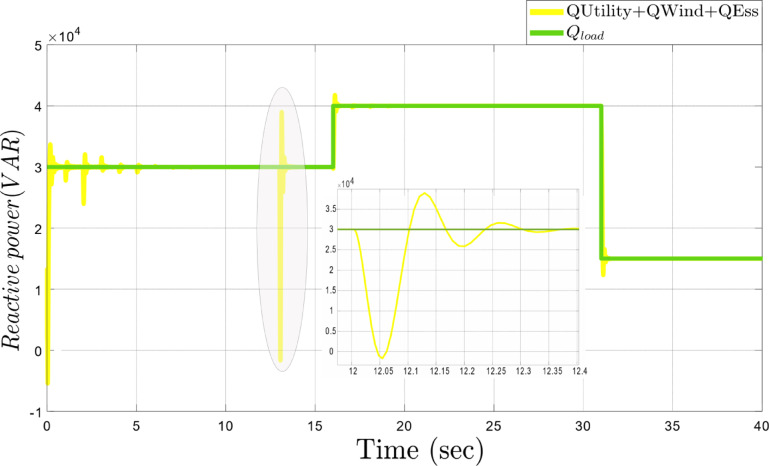
Dynamic response of the proposed framework to sudden reactive power changes.

According to Fig. 6, the efficacy of the suggested strategy is checked for reactive power fluctuations of both increases and decreases in magnitude. At the 15th second, the reactive power rises from 30 kVAR to 40 kVAR, then at the 31 st second, it decreases to 5 kVAR. This simulation shows the ability of the framework to respond well to reactive power variations. At the 12th second, as shown in the zoom area of Fig. 6, there is a sudden drop in reactive power because of a disturbance caused by an increase in active power demand. In normal conditions or during gradual changes, active power management is straightforward, using all four degrees of freedom: Inverter 1, Inverter 2, Inverter 3, and the microturbine. However, this event limits reactive power control to the three inverters since the microturbine is unavailable for reactive power regulation. Despite these constraints, the system stabilizes active power rapidly and controls reactive power fluctuations effectively. These minor fluctuations, resolved within 0.05 s, are insignificant and do not compromise overall system performance. This demonstrates the resilience and adaptability of the proposed framework in handling sudden power variations.

**Fig. 7:**
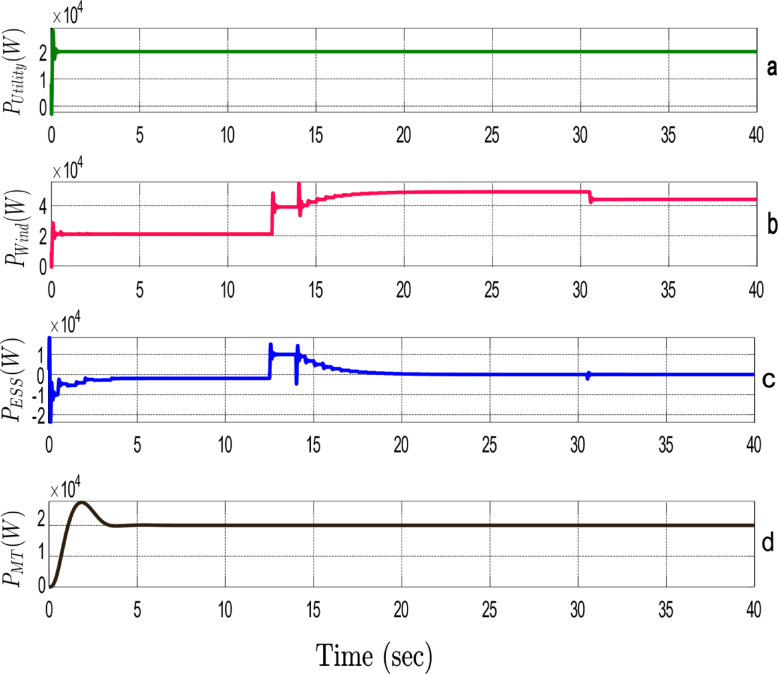
Power-sharing dynamics among DERs in response to varying active power demands in the grid-connected MG.

Figure 7 shows the power-sharing dynamics among all the DERs. If the network experiences a sudden rise in active power demand, then the system quickly detects the requirement to supply more power. Inverter 2, representing the wind turbine (Fig. 7-b), and inverter 3, representing the ESS (Fig. 7-c), immediately start supplying power to the grid. In the proposed framework, the decision-making criteria for power allocation among DERs are based on two major factors: technical parameters and economic parameters. During high power demand periods, the system selects the most cost-effective and technically efficient resources. Therefore, the wind turbine and ESS are activated because their generation costs are lower than the utility, which ensures the optimal balance between economic efficiency and technical performance. The quick response of the ESS to sudden changes in demand, along with the wind turbine and cost-effective electricity generation render them the first-choice resources of the system. This behavior is clearly depicted in Fig. 7-a. one of the main goals of the framework is to minimize dependence on the main grid, ensuring that the MG can meet increasing demand primarily through internal resources. Furthermore, Fig. 7-d illustrates the contribution of the microturbine, which continuously injects constant active power into the network to help regulate active power during fluctuations. Acting as an additional degree of freedom, the microturbine provides flexibility to the system, ensuring stable power delivery and supporting the overall power-sharing strategy during periods of demand variability.

**Fig. 8:**
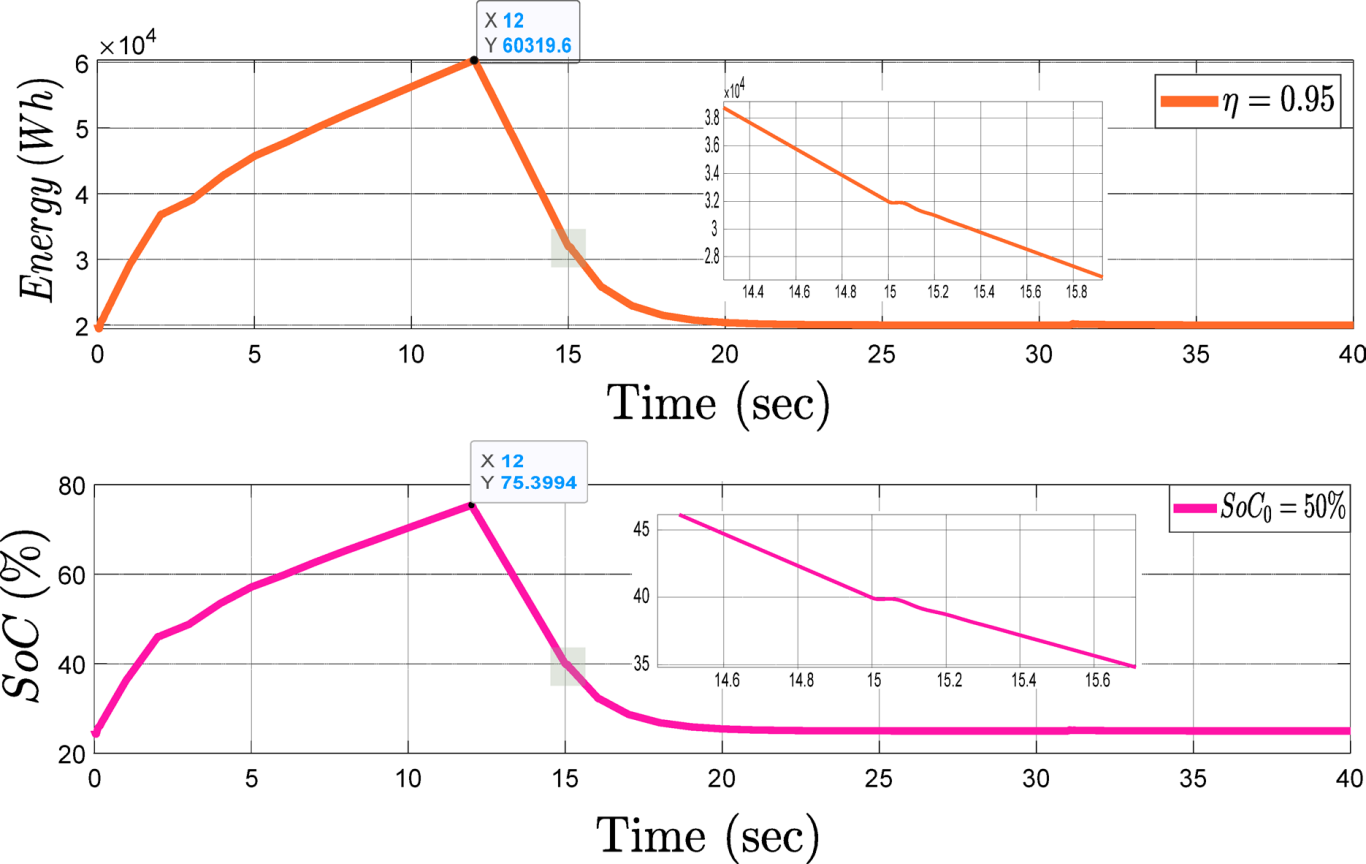
Charge and discharge process of the ESS within the proposed framework.

The proposed framework for managing ESS employs a protective range between 25% and 80% in order to prevent deep discharge of the battery. When the battery state of charge falls below 25%, there is a greater risk of chemical degradation. Discharging the battery to this level can reduce its capacity and lifespan, and its efficiency can decrease, making it unable to supply the required power effectively. On the other hand, charging beyond 80% may force the chemical structure of the battery and raise its temperature, thus causing serious damage or safety risks like explosion and fire. Therefore, the protective ranges from 25 to 80% have been established in order to maintain the longevity and efficiency of the battery. Figure 8 shows the charge/discharge process of the battery storage in this network. Before the 12th second, the battery is in charge mode and, as depicted, acts as a bus grid-following bus. At the 12th second, the network experiences a sudden rise in active power. The battery storage supplies the active power demand and acts as a grid-coordinated bus. As clearly shown in the zoomed-in section of the figure, at the 15th second, when the network requires an increase in reactive power, the battery becomes online to supply the reactive power and increases its discharge rate to provide active and reactive power to the network. At the 20th second, because of the protection limits set on the ESS, the battery ceases to inject power into the grid when its state of charge reaches 25% of its capacity.

**Fig. 9:**
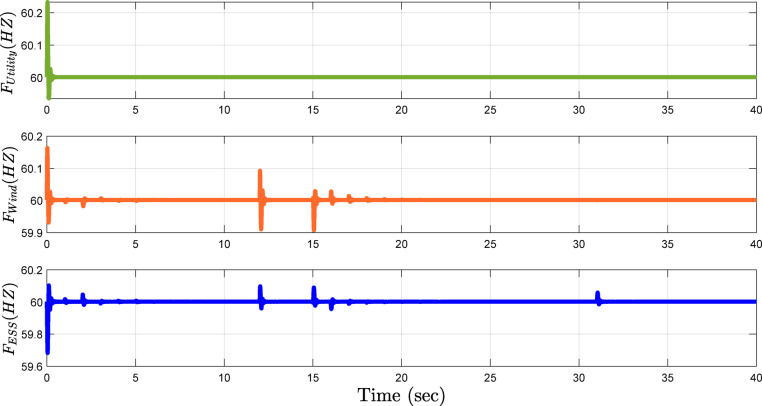
Frequency stability of the MG under varying active and reactive power conditions.

One of the main objectives of this study is stability in frequency. Figure 9 illustrates that the proposed framework effectively controls the frequency during fluctuations in active power, reactive power, or in response to uncertainties in wind speed, which highly influences the rate of power generation. As a result of these changes, the system frequency remains steady, showcasing the potential of the framework in balancing both the technical and economic capabilities of the microgrid. Figure 9 depicts the frequency of the utility as remaining within 60 Hz, while variations within the active and reactive power cause slight increases in the frequencies of the wind turbine and ESS inverters, respectively. However, these fluctuations are in the range of less than 0.01 Hz, which indicates the excellent performance of the proposed approach.

**Fig. 10:**
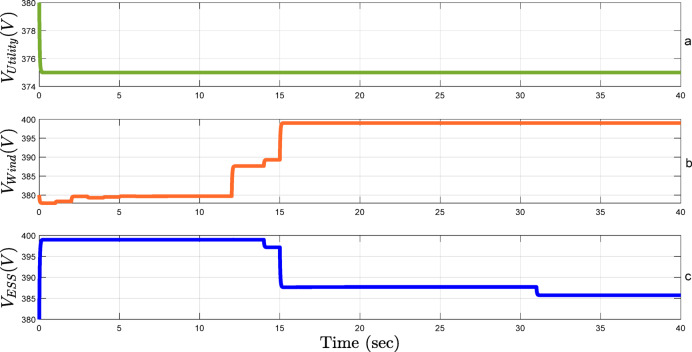
Voltage stability of the MG under fluctuations in active and reactive power.

Another main objective of this study is to keep voltage stability. In the setup under study, the nominal voltage is set as 380 V. Figure 10 shows that the proposed framework effectively regulates voltage under fluctuations due to changes in active power, reactive power, or uncertainties in wind speed, which highly influences the rate of power generation. Regardless of these fluctuations, the voltage of the system remains constant at around 375 V in the utility, as indicated by the green line in Fig. 10-a. The wind turbine inverter plays a crucial role in voltage control. The voltage remains constant and steady at 380 V, without any fluctuations from 0 to 12 s. However, the active power consumption of the system increases suddenly at the 12th second. Accordingly, the voltage across the network is adjusted by the wind turbine inverter, which momentarily rises up to around 390 V. Later, in the 15th second, there is a severe increase in reactive power consumption in the system. In response to this, the wind turbine inverter adjusts once again in order to keep voltage stability, so the voltage rises to around 399 V, which is shown in Fig. 10-b. The proposed framework for system control in inverters and ESS is designed to operate in a coordinated manner, responding dynamically to grid changes. As shown in Fig. 10-b, when the voltage in the wind turbine inverter increases between 12 and 15 s, when the voltage in the wind turbine inverter increases, the ESS adjusts its voltage downwards to dampen the voltage fluctuations in the network, as seen in Fig. 10-c. This behavior highlights the effectiveness of the proposed framework in maintaining grid balance and stability. Additionally, at the 31 st second, when both active and reactive power consumption decrease, the ESS inverter gradually approaches its nominal setpoint value.

**Fig. 11:**
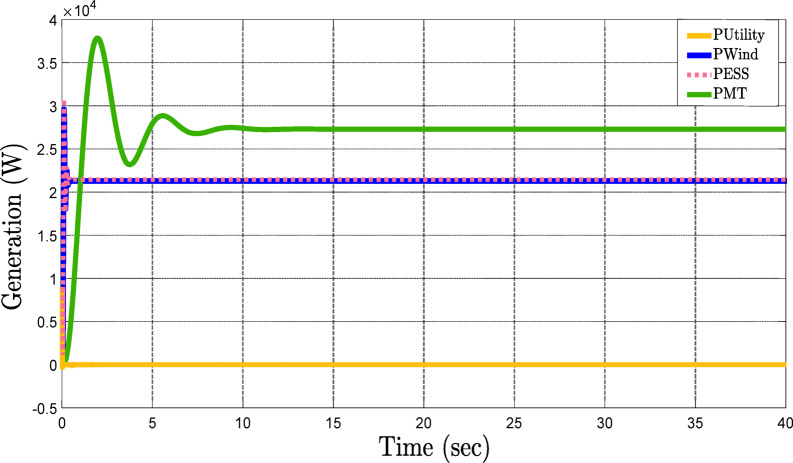
Scalability and power-sharing performance of the proposed framework tested on a 9-bus IEEE network.

Another key objective of this study is to assess the scalability of the proposed framework when applied to larger networks. The framework is tested on IEEE 9-bus, with the key performance indices, EENS, power loss, and voltage deviation. The results obtained can be further extended to larger networks. Figure 11 shows that the MG meets the power consumption of the network without drawing power from the main grid. By fully utilizing the capacity of the MG, this approach not only maximizes system performance but also reduces costs by getting rid of the need for buying energy from external sources. This shows the potential of the framework for better efficiency and cost-effectiveness for larger systems.

**Fig. 12:**
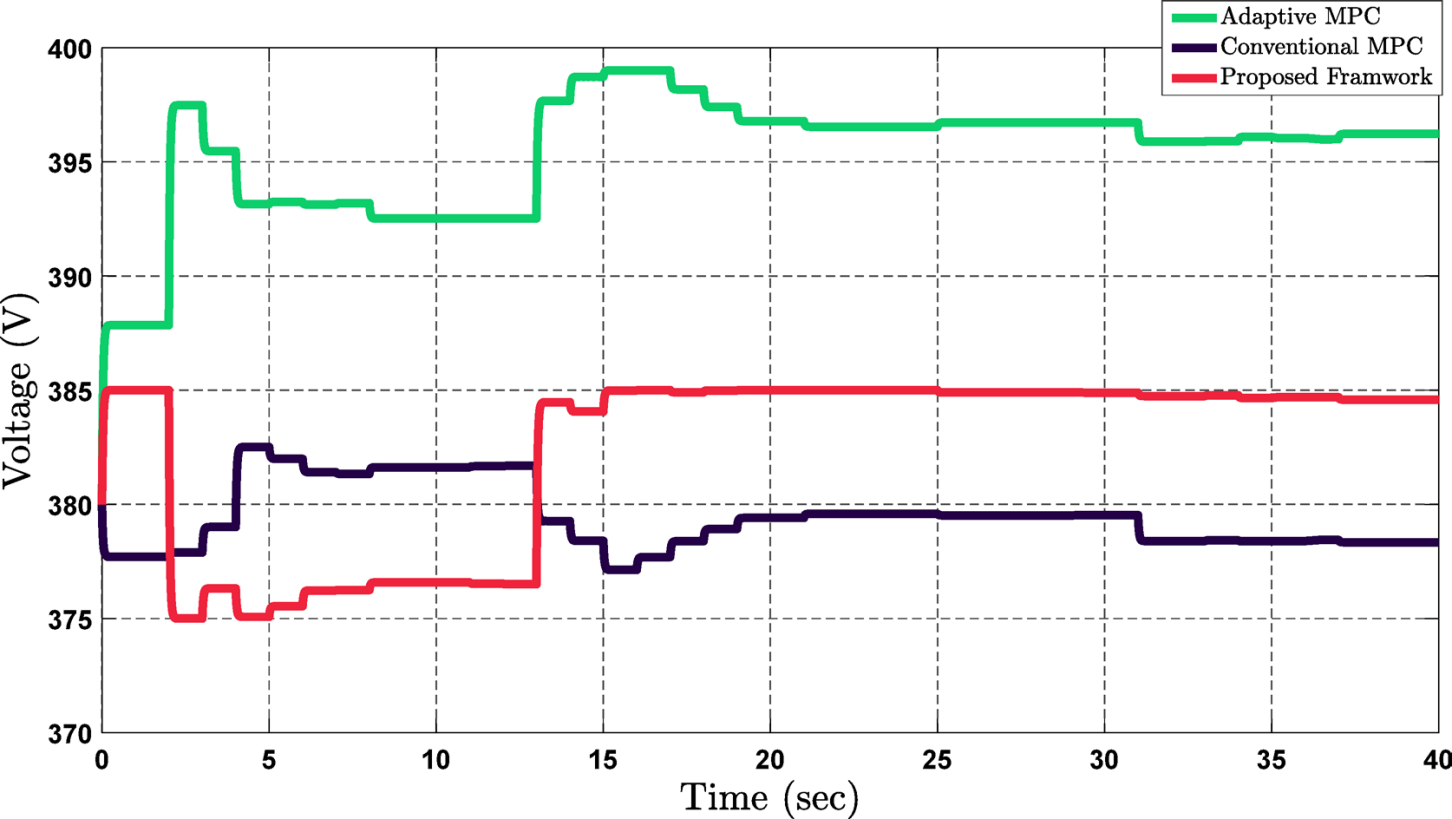
Voltage stability comparison of the proposed framework with Adaptive MPC and Conventional MPC under active and reactive power fluctuations.

This research evaluated the proposed framework through a comparison with Conventional MPC^44,45^ and Adaptive MPC^46,47^. The first comparison evaluated how the proposed framework performs in terms of overall technical aspects before and after fluctuations in active and reactive power demand. As shown in Fig. 12, under these circumstances, voltage stability decreased and fluctuated in Conventional MPC, exhibiting an average percentage deviation of 1.82% lower, with a minimum and maximum range of 373 to 383 volts. On the contrary, within Adaptive MPC, the voltage increased and fluctuated around a range of 393 to 398 volts, with an average percentage deviation of 2.73% increase. This result shows the adaptability of the proposed framework with respect to the unstable conditions for maintaining voltage stability. As depicted in Fig. 12, the framework proposed effectively maintains the voltage to its nominal value of 385 Volts in fluctuations of active and reactive power demand. The proposed framework showed a 28.57% improvement over Conventional MPC and a 52.38% improvement over Adaptive MPC.

**Fig. 13:**
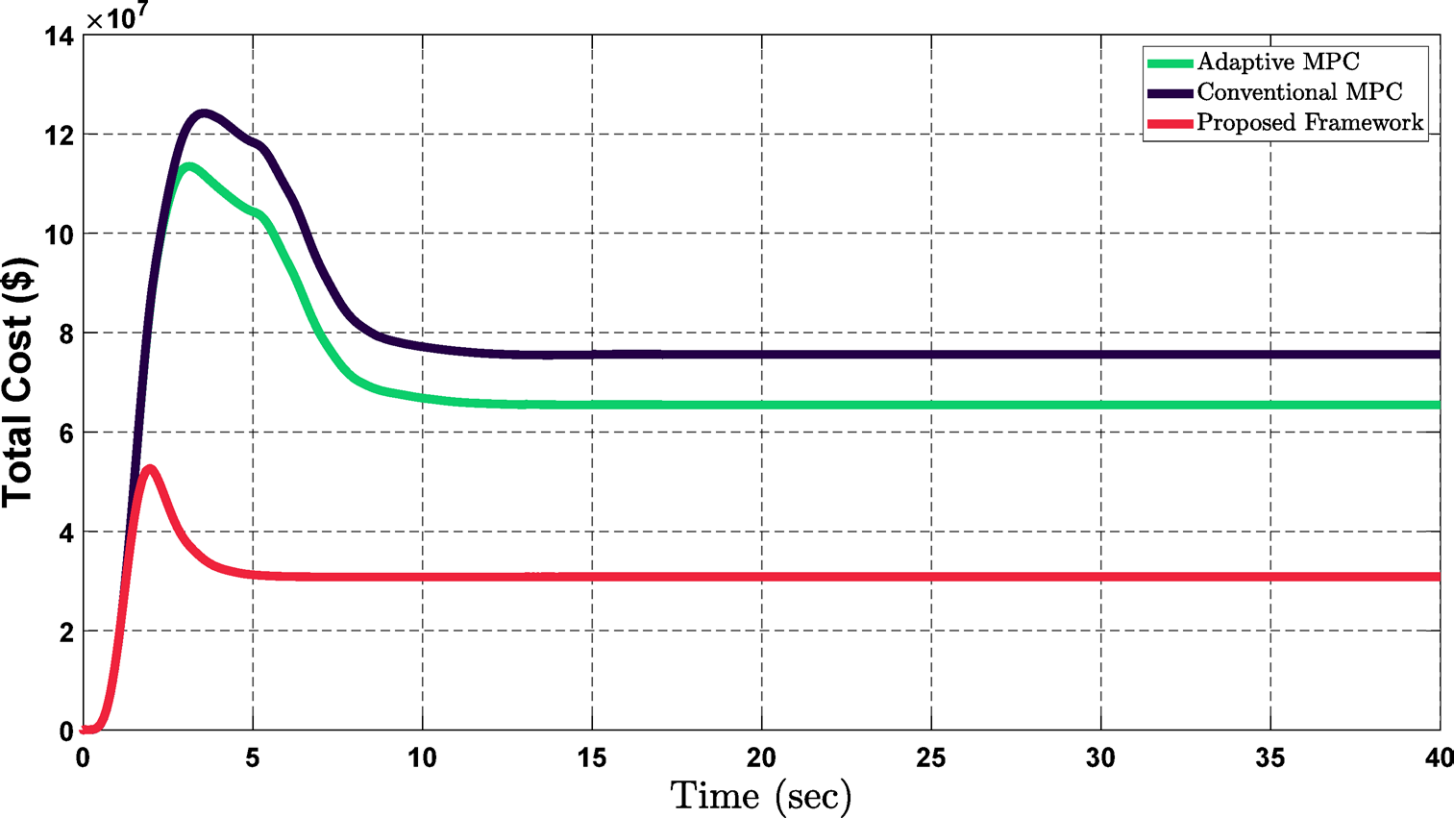
Operational cost comparison of the proposed framework with Adaptive MPC and Conventional MPC, highlighting cost savings during power fluctuations.

The superiority of the proposed framework in economic aspects is addressed in the following comparison. In order to define the economic advantages, it offers, it can be seen in Fig. 13 that the framework has enhanced the operational costs of the network during fluctuations in the active and reactive power demand. One clear reason for this is that the suggested structure is independent of the main grid, which helps it minimize operational costs in comparison with Conventional MPC^44,45^ and Adaptive MPC^46,47^. Another factor highlighting the superiority of the offered structure is that it is able to preserve stability even during a startup phase; this phase is shorter for the proposed GPR-based real-time decisions microgrid optimization since it reaches a steady state much quicker than Conventional MPC and Adaptive MPC have shortcomings at this stage. Therefore, the proposed framework achieves almost 39.2% cost savings in comparison with the Conventional MPC and approximately 41.5% relative to the Adaptive MPC system.

In this study, the proposed framework has been compared with Conventional MPC^44,45^ and Adaptive MPC^46,47^ using Figs. 12 and 13, which focused on voltage deviation and operation cost. To further enhance the credibility of the proposed framework, Table 3 presents a quantitative comparison between the proposed framework and other studies.

**Table 3:** Comparative evaluation of the proposed method with existing works.

Reference	Voltage deviation (VD) [%]	Frequency deviation (FD) [%]	Total Operation cost [$]
^32^– Uncertainty-Aware ESS	2.6–4.7	0.05–0.083	4.2 × 10⁷
^35^– GPR for PV Inertia	2.1–4.0	0.042–0.067	4.0 × 10⁷
^36^– CSMOBA with Wind Uncertainty	1.8–3.3	0.034–0.059	3.7 × 10⁷
^37^– Hybrid Data-Driven Reliability	1.6–3.0	0.030–0.050	3.5 × 10⁷
Proposed Framework	< 1.3	< 0.017	3.1 × 10⁷

According to Table 3, the proposed framework demonstrates improved performance on all three criteria. Based on voltage deviation (VD), the proposed framework maintains VD below 1.3%, which is extremely lower than the ranges reported in previous studies (1.6–4.7%). This reflects enhanced voltage stability, owing to the employed predictive control and inverter coordination mechanism. As for frequency deviation (FD), the proposed framework maintains FD within 0.017%, improved in comparison to existing works where FDs range from 0.03 to 0.08%, showing robustness of the suggested framework under uncertainty and variable operating conditions. For the total operating cost, the proposed strategy achieves the lowest cost outcome at approximately 3.1 × 10⁷ USD, which is at least 11% lower than the next best model and significantly better compared to others. The cost advantage results from the accurate forecasting of DGs and ESS operation using GPR, optimal power flow control, and minimizing dependence on the main grid. These findings confirm the technical and economic advantage of the proposed framework.

## Conclusions

The present investigation addresses the critical need for sustainable solutions in response to rising energy demands by an innovative data-driven NLMPC strategy for grid-connected MGs. This strategy enhances operational performance with the integration of both rotating and non-rotating DG resources such as wind turbines, microturbines, and battery storage while ensuring voltage and frequency stability. The results indicate that the suggested framework outperforms traditional methods in voltage stability by 28.57%, while the overall cost-saving is about 39.2% in comparison to Conventional MPC and around 41.5% compared to Adaptive MPC. Gaussian Process Regression ensures a framework capable of handling such challenges under dynamic operating conditions due to the ability to achieve an exact prediction and use Monte Carlo simulations to manage uncertainty. Besides, the dynamic interaction between ESS and other inverters highlights the responsiveness of the framework for active and reactive power variations with regard to technical-economic efficiency. Protective measures implemented for battery management further enhance the reliability and longevity of the system. Further analysis confirms the scalability of the proposed framework, demonstrating its effectiveness in managing larger systems, as evidenced by the IEEE 9-bus network. This capability allows it to meet power demands without depending on the main grid. In addition, the ability of the strategy to maintain voltage within nominal ranges despite fluctuations in consumption underlines the robustness of the proposed control strategy. The proposed framework advances the efficiency and cost-effectiveness of microgrid operations while laying the groundwork for future research work in optimizing larger-scale energy systems. This work paves the way for more resilient and sustainable energy infrastructures, which are necessary to face contemporary energy challenges.

Future research should focus on expanding the scalability of the proposed data-driven NLMPC framework to larger and more complex power networks, ensuring its robustness in real-world applications. Integrating advanced AI techniques, such as deep reinforcement learning and hybrid models, could further enhance adaptive decision-making under dynamic grid conditions. Additionally, cybersecurity measures must be explored to protect microgrid operations from potential cyber threats, ensuring resilience and reliability. Another important direction is optimizing multi-microgrid coordination to improve energy efficiency and system resilience. Incorporating renewable energy market dynamics and demand-side management strategies within the framework can enhance its economic feasibility. Moreover, improving battery degradation modeling will provide better insights into long-term energy storage performance, contributing to more sustainable and cost-effective microgrid management. Addressing these areas will further refine the proposed strategy, making it more adaptable, resilient, and applicable to future energy systems.

## Data Availability

The datasets used and/or analysed during the current study available from the correspondingauthor on reasonable request.

## Abbreviations

DER: Distributed Generation Resources
DSOs: Distribution System Operators
EENS: Expected Energy Not Supplied
MGs: Microgrids
MPC: Model Predictive Control
PL: Power Loss
SOC: State-Of-Charge
LAL: Load Ability Limited
*y(t)*: Optimize System Outputs
*u(t)*: Control Inputs
$$\wp$$: Uncertainties
$\Im_{GPR}$: System Dynamics Modeled Using GPR
H: Uncertainty Scenarios
$u^{*}_{t}$: Optimal Control Input
$$\theta$$: Phase Angle
*A*: Swept Area of The Wind Turbine Rotor
$\ell$: Function Of Tip-Speed Ratio
$Cos\:t^{ESS}_{capital}$: Capital Cost of ESS
$\rho^{ESS} (t)$: Charging or Discharging Capacity of ESS
DG: Distributed Generation
ESS: Energy Storage System
GPR: Gaussian Process Regression
MMO: Multi-Input Multiple-Output
NLMPC: Nonlinear Model Predictive Control
NPM: Network-Preserving Models
RES: Renewable Energy Sources
TSOs: Transmission System Operators
VD: Voltage Deviation
*x(t)*,: State Variables
$$\Im$$: System Dynamics
$\Im_{WT}$: GPR Model Predicts the Output Power of Wind Turbine
$$U$$: Voltage
$$\gamma$$: Air Density
$$\beta$$: Pitch Angle
$$V_{wind}(t)$$: Wind Speed
C_ESS_: Capacity of the ESS
$\rho^{ESS}_{capital}$: Investment Cost Per Unit of Capacity for ESS

## References

[1] Iweh, C. D., Gyamfi, S., Tanyi, E. & Effah-Donyina, E. Distributed generation and renewable energy integration into the grid: prerequisites, push factors, practical options, issues and merits. *Energies* **14** (17), 5375 (2021).

[2] Ullah, Z. et al. Advanced energy management strategy for microgrid using real-time monitoring interface. *J. Energy Storage*. **52**, 104814 (2022).

[3] Bandeiras, F., Gomes, Á., Gomes, M. & Coelho, P. Exploring energy trading markets in smart grid and microgrid systems and their implications for sustainability in smart cities. *Energies* **16** (2), 801 (2023).

[4] Kiehbadroudinezhad, M. et al. The role of energy security and resilience in the sustainability of green microgrids: paving the way to sustainable and clean production. *Sustain. Energy Technol. Assess.* **60**, 103485 (2023).

[5] Jahromi, M. Z., Yaghoubi, E. & Yaghoubi, E. Optimal generation and distribution planning in smart microgrids under conditions of multi-microgrid Disconnection using a hierarchical control strategy. *Electrical Engineering*, 1–20 10.1007/s00202-025-03036-4 (2025).

[6] Maghami, M. R. et al. Hybrid energy management with respect to a hydrogen energy system and demand response. *Int. J. Hydrog. Energy*. **45** (3), 1499–1509 (2020).

[7] Taghikhani, M. A. Renewable resources and storage systems stochastic multi-objective optimal energy scheduling considering load and generation uncertainties. *J. Energy Storage*. **43**, 103293 (2021).

[8] Maghami, M. R., Pasupuleti, J. & Ling, C. M. A static and dynamic analysis of photovoltaic penetration into MV distribution network. *Processes* **11** (4), 1172 (2023).

[9] Arévalo, P., Ochoa-Correa, D. & Villa-Ávila, E. Optimizing microgrid operation: integration of emerging technologies and artificial intelligence for energy efficiency. *Electronics* **13** (18), 3754 (2024).

[10] Razmi, D. et al. Review of model predictive control of distributed energy resources in microgrids. *Symmetry* **14** (8), 1735 (2022).

[11] Safaei Pirooz, A. A. et al. Adaptation of high spatio-temporal resolution weather/load forecast in real-world distributed energy-system operation. *Energies* **16** (8), 3477 (2023).

[12] Jahromi, M. Z., Yaghoubi, E., Yaghoubi, E., Maghami, M. R. & Chamorro, H. R. An innovative Real-Time recursive framework for Techno-Economical Self-Healing in large power microgrids against Cyber–Physical attacks using large change sensitivity analysis. *Energies* **18** (1), 190 (2025).

[13] Khather, S. I., Ibrahim, M. A. & Abdullah, A. I. Review and performance analysis of nonlinear model predictive Control–Current prospects, challenges and future directions. *Journal Européen Des. Systèmes Automatisés* **56**(4), 593–603 10.18280/jesa.560409(2023).

[14] Keivanimehr, M. et al. A hybrid method based on corrected kinetic energy and statistical calculation for Real-Time transient stability evaluation. *Processes* **12** (11), 2409 (2024).

[15] Shezan, S. A. et al. Evaluation of different optimization techniques and control strategies of hybrid microgrid: A review. *Energies* **16** (4), 1792 (2023).

[16] Gao, J., Maalla, A., Li, X., Zhou, X. & Lian, K. Comprehensive model for efficient microgrid operation: addressing uncertainties and economic considerations. *Energy* **306**, 132407 (2024).

[17] Hussain, W. Strategic Decision-Making in uncertain environments: A management science perspective. *Manage. Sci. Letter*. **1** (3), 64–77 (2024).

[18] Khan, M. A., Bayati, N. & Ebel, T. Techno-economic analysis and predictive operation of a power-to-hydrogen for renewable microgrids. *Energy. Conv. Manag.* **298**, 117762 (2023).

[19] Chen, Y., Ji, C., Cai, Y., Yan, T. & Su, B. Deep Reinforcement Learning in Autonomous Car Path Planning and Control: A Survey, *arXiv preprint arXiv:2404.00340*, (2024).

[20] Spiegel, M. H., Veith, E. M. & Strasser, T. I. The spectrum of proactive, resilient multi-microgrid scheduling: A systematic literature review. *Energies* **13** (17), 4543 (2020).

[21] Negi, G. S. et al. Empowering sustainability and resilience: the prominence of microgrids in a decentralized energy future. *ICSCSS*, 25–31 (2024).

[22] Yaghoubi, E., Yaghoubi, E., Maghami, M. R. & Jahromi, M. Z. Comprehensive technical risk indices and advanced methodologies for power system risk management. *Electr. Power Syst. Res.* **244**, 111534 (2025).

[23] Morato, M. M., Normey-Rico, J. E. & Sename, O. Model predictive control design for linear parameter varying systems: A survey. *Annu. Rev. Control.* **49**, 64–80 (2020).

[24] Shen, F., Zhao, L., Du, W., Zhong, W. & Qian, F. Large-scale industrial energy systems optimization under uncertainty: A data-driven robust optimization approach. *Appl. Energy*. **259**, 114199 (2020).

[25] Geng, S., Wu, G., Tan, C., Niu, D. & Guo, X. Multi-objective optimization of a microgrid considering the uncertainty of supply and demand. *Sustainability* **13** (3), 1320 (2021).

[26] Sharma, T. & He, Y. Design of a tracking controller for autonomous articulated heavy vehicles using a nonlinear model predictive control technique, *Proceedings of the Institution of Mechanical Engineers, Part K: Journal of Multi-body Dynamics*, p. 14644193241232353, (2024).

[27] Procter, A. *Demand Led Tidal Lagoon Power and Hydrogen Energy Storage-Supervisory Control and Optimisation* (University of South Wales (United Kingdom), 2022).

[28] Abir, S. A. A., Anwar, A., Choi, J. & Kayes, A. Iot-enabled smart energy grid: applications and challenges. *IEEE Access.* **9**, 50961–50981 (2021).

[29] Aslam, S. et al. A survey on deep learning methods for power load and renewable energy forecasting in smart microgrids. *Renew. Sustain. Energy Rev.* **144**, 110992 (2021).

[30] Mandloi, T., Sharma, S. K. & Choube, S. Energy management in microgrid employing unit commitment considering diverse system uncertainties. *Electrical Engineering*107 2487–2505 10.1007/s00202-024-02651-x (2025).

[31] Ferdaus, M. M., Al-Mahasneh, A. J., Anavatti, S. G. & Senthilnath, J. A compact meta-learned neuro-fuzzy technique for noise-robust nonlinear control. *Appl. Soft Comput.* **166**, 112149 (2024).

[32] Reza, M. et al. Uncertainty parameters of battery energy storage integrated grid and their modeling approaches: A review and future research directions. *J. Energy Storage*. **68**, 107698 (2023).

[33] Najibi, F. *Enhanced Power System Operation with Coordination and Forecasting Techniques* ( City, University of London, 2021).

[34] Dahmani, S. Energy optimization and smart grids: IoT-Based smart grid solution and smart grids applications, Harnessing High-Performance Computing and AI for Environmental Sustainability. IGI Global, 278–304 10.4018/979-8-3693-1794-5.ch013 (2024).

[35] Kanwal, S., Khan, B., Ali, S. & Mehmood, C. Gaussian process regression based inertia emulation and reserve Estimation for grid interfaced photovoltaic system. *Renew. Energy*. **126**, 865–875 (2018).

[36] Sun, S., Wang, C., Wang, Y., Zhu, X. & Lu, H. Multi-objective optimization dispatching of a micro-grid considering uncertainty in wind power forecasting. *Energy Rep.* **8**, 2859–2874 (2022).

[37] Zhang, S. et al. Combing data-driven and model-driven methods for high proportion renewable energy distribution network reliability evaluation. *Int. J. Electr. Power Energy Syst.* **149**, 108941 (2023).

[38] Sakki, G. K., Tsoukalas, I., Kossieris, P., Makropoulos, C. & Efstratiadis, A. Stochastic simulation-optimization framework for the design and assessment of renewable energy systems under uncertainty. *Renew. Sustain. Energy Rev.* **168**, 112886 (2022).

[39] Wang, G., Verleysen, K., De Meulenaere, R. & Blondeau, J. Multi-objective optimization of hybrid energy storage systems under uncertainty. *J. Energy Storage*. **111**, 115218 (2025).

[40] Zhang, N., Xiong, J., Zhong, J. & Leatham, K. Gaussian process regression method for classification for high-dimensional data with limited samples. *IEEE* 358–363 10.1109/ICIST.2018.8426077 (2018).

[41] Deng, Y., Eden, M. & Cremaschi, S. A Gaussian process embedded feature selection method based on automatic relevance determination. *Comput. Chem. Eng.* **191**, 108852 (2024).

[42] Bagchi, S., Bhowmik, P., Chakraborty, R. & Das, P. Optimal bandwidth-based pseudo-centralized droop control mechanism for grid-forming microgrids using tri-layered neural network with real-time feasibility. *Electrical Engineering* **107**, 7137–7156 10.1007/s00202-024-02926-3 (2024).

[43] Yang, M., Wang, J., Zhou, Y., Han, G. & Kang, J. Optimal configuration of integrated energy system based on multiple energy storage considering source-load uncertainties under different risk tendencies. *J. Energy Storage*. **109**, 115220 (2025).

[44] Ke, S. et al. A frequency control strategy for EV stations based on MPC-VSG in islanded microgrids. *IEEE Trans. Industr. Inf.* **20** (2), 1819–1831 (2023).

[45] Vidyasagar, P. & Shanti Swarup, K. LTI-MPC for the Micro-grid control, Design and Development of Model Predictive Primary Control of Micro Grids: Simulation Examples in MATLAB. Springer, 69–90 (2023).

[46] Feng, K. & Liu, C. Adaptive DMPC-based frequency and voltage control for microgrid deploying a novel EV-based virtual energy router. *IEEE Trans. Transp. Electrification*. **10** (3), 4978–4989 (2023).

[47] Omran, A. S., Hamad, M. S., Abdelgeliel, M. & Abdel-Khalik, A. S. An adaptive model based on data-driven approach for FCS-MPC forming converter in microgrid. *Int. J. Control Autom. Syst.* **21** (11), 3777–3795 (2023).

